# Chromogenic Anticounterfeit and Security Papers: An Easy and Effective Approach

**DOI:** 10.1021/acsami.1c19228

**Published:** 2021-12-07

**Authors:** José Carlos Guirado-Moreno, Marta Guembe-García, José M. García, Roberto Aguado, Artur J. M. Valente, Saúl Vallejos

**Affiliations:** † Departamento de Química, Facultad de Ciencias, Universidad de Burgos, Plaza de Misael Bañuelos s/n, 09001 Burgos, Spain; ‡ CQC, Department of Chemistry, University of Coimbra, Rua Larga, 3004-535 Coimbra, Portugal

**Keywords:** Anticounterfeit, papers, azo-coupling, sensory polymers, colorimetry, RGB parameters

## Abstract

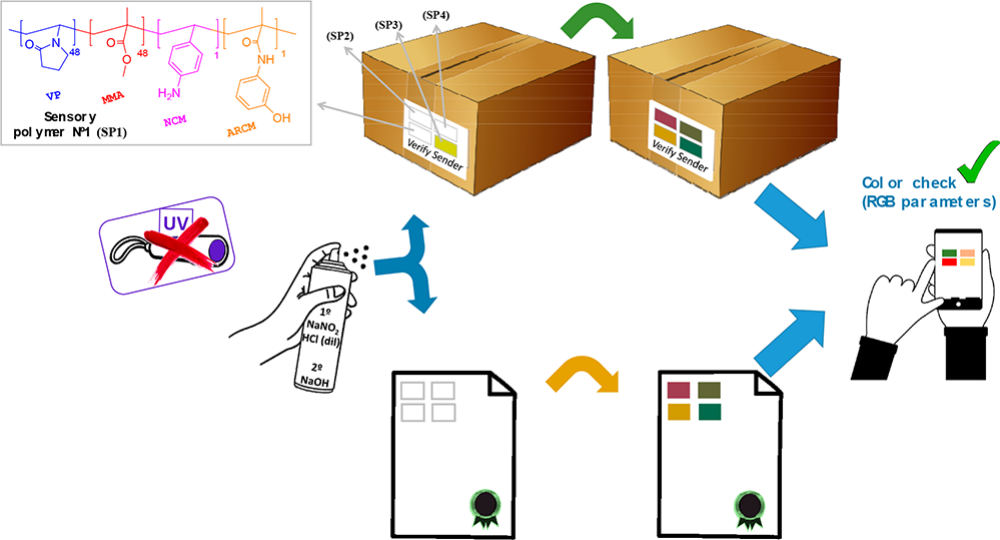

The synthesis and preparation of 12 chromogenic polymers used to build an intelligent label for security paper applications are described. The process involves coating paper sheets with the polymers. Depending on the number of different polymers used in a combinatory way, a maximum of 12^12^ combinations is possible, thus creating a matrix that is practically impossible to counterfeit. Currently, most anticounterfeiting proposals for paper-based packaging and documents involve some sort of verification under ultraviolet radiation, and the requirement of additional equipment often relegates the end-user to a passive role. In contrast, in our approach, the combination of sensory polymers in an array gives rise to an invisible label, i.e., an owner cryptographic key, which becomes visible upon scattering a nitrite solution (e.g., spraying or using an impregnated foam roller) over the printed label on the security paper. For this purpose, a monomer containing an aromatic primary amino group and another with an activated aromatic ring are covalently bonded to a polymer with high affinity toward paper, consisting essentially of units of methyl methacrylate and 1-vinyl-2-pyrrolidone. Subsequently, the paper samples are coated with the resulting sensory chromogenic polymer. By spraying, painting, or staining an aqueous acid solution of NaNO_2_ (at least 1.20 g/L) and the chromogenic polymers, a well-defined color appears, because of the formation of an azo compound. This process provides users with a quick and facile authentication method without additional equipment and without affecting paper strength.

## 1 Introduction

Counterfeiting harms not only many industrial sectors but also different levels of society, from deceived final users to public administrations.^1^ Alarmingly, global losses because of this illegal activity are expected to reach between 1.6 and 2.3 trillion USD in 2022,^2^ a fair part of that going straight into the coffers of criminal organizations.^3^ Faced with such a big threat, anticounterfeiting techniques have been a widely studied scientific field over the last few decades.

More specifically, in the frame of security papers, most works are based on luminescent security elements, as inks, carbon dots, or markers.^4−10^ However, UV–B or UV–C irradiation, rarely at the disposal of the end-user, is often necessary for activating or revealing luminescent materials. Furthermore, the frequent use of metal complexes as fluorophores increases the price of the product and converts the paper to a hybrid product with both organic and inorganic parts, which affects its safety and sustainability. Other proposals require barcode readers or other types of scanning, but common smartphones can be used as well, once certain software is installed.^11,12^

A source of inspiration comes from colorimetric sensor arrays, i.e., groups of sensory materials distributed over a substrate in a certain pattern so that each material undergoes a change of color in the presence of a specific target, with more or less selectivity.^13−17^ There are many well-known possibilities to attain that. For instance, 3,3′,5,5′-tetramethylbenzidine changes from colorless to blue upon oxidation,^14^ and its immobilization in paper has already been proven successful.^18^ Nanoparticles of gold, silver, and even copper, whose mechanism of colorimetric detection is surface plasmon resonance, have likewise been used as a coating layer for paper-based immunosensors.^18^ However, these and other approaches, such as metalloporphyrins, cobalt(II) chloride, or colorimetric Cd–Te quantum dots,^19^ generally require heavy-metal ions, be it as the analyte that triggers the response, as part of the sensing system itself, or both. Furthermore, the number of possible colors is rather limited in most cases. Likely, the sensor array that resembles our approach the most is Park et al.’s organic solvatochromic system, which results in different combinations of colors when exposed to different organic compounds.^20^ With that principle in mind, but shifting the aim from pollutant detection to authentication, azo compounds not only provide a broader range of colors, but also do not require volatile organic solvents to trigger the response.

All considered, we have designed a new verification technique that provides an evident visual response without the need for additional equipment, although a smartphone can aid users in identifying every color with accuracy. First, the dispatcher defines a certain pattern or sequence of colors. This colored code is hidden as colorless and/or yellowish (public-key encryption) by printing, painting, or coating on paper several fully organic copolymers, each containing small proportions of different sensory units. The final user has only to spray an inexpensive solution, and the array of sensory polymers, which comprise the anticounterfeiting system, changes its color under visible light (private key decryption), making any excitation with UV radiation unnecessary.

The sensory copolymers are mainly based on commercially available 1-vinyl-2-pyrrolidone (VP) and methyl methacrylate (MMA), granting high compatibility with paper and ensuring that there is no elution when the verification solution is applied. These copolymers are chromogenic, because of small amounts of both a monomer containing an aromatic amine group (MCN), which is susceptible to forming a derivative of benzene diazonium salt, and an activated ring-containing monomer, e.g., phenols, *N*,*N*-dimethylaminoanilines, and the like (MCAR). Only when the verification solution (made with reagents available in common hardware stores) is sprayed, painted, or stained over the paper, the azo coupling reaction between both sensory motifs (MCN and MCAR) starts, and the colored azo dye is formed.^21−23^ Optionally, applying an alkaline solution (also available in hardware stores) prompts the reaction and results in an immediate response. We have prepared 12 different polymers combining 3 MCN and 4 MCAR. The possibility of obtaining different colors, one per polymer, allows the dispatcher to provide customers with numerous chromatic codes.

## 2 Experimental Section

### 2.1 Materials

All materials and solvents were commercially available and used as received unless otherwise indicated. The following materials and solvents were used: methyl methacrylate (MMA) (Merck, 99%), 1-vinyl-2-pyrrolidone (VP) (Acros Organic, 99%), 4-aminostyrene (SNH_2_) (TCI, 98%), hydrochloric acid (VWR-Prolabo, 37%), sodium hydroxide (VWR-Prolabo, 99%), *N*,*N*′-dicyclohexylcarcodiimide (Aldrich, 99%), tetrahydrofuran (VWR-Prolabo, 99.9%), ethanol (VWR-Prolabo, 99.9%), dichloromethane (VWR-Prolabo, 99.9%), 4-amino-1,8-naphtalic anhydride (Merck, 95%), hydrazine monohydrate (Panreac, 80%), triethylamine (VWR, 99%), methacryloyl chloride (Alfa Aesar, 97%), 4-aminobenzyl alcohol (Merck, 98%), ethyl acetate (VWR-Prolabo, 99.9%), methacrylic anhydride (Alfa Aesar, 94%), manganese oxide activated (Fluka, 90%), Celite 503 (Merck), aniline (Alfa Aesar, 99%), methanol (VWR-Prolabo, 99.9%), sodium borohydride (Alfa Aesar, 98%), paraformaldehyde (Aldrich, 95%), trifluoroacetic acid (Alfa Aesar, 99.5%), sodium hydroxide (VWR-Prolabo, 99%), sodium chloride (VWR-Prolabo, 98%), 1-boc-piperazine (Merck, 97%), diethyl ether (VWR-Prolabo, 99.9%), 1,4-dioxane (VWR, 99.9%), potassium carbonate (VWR-Prolabo, 99%), sodium sulfate anhydrous (VWR-Prolabo, 99%), hexane (VWR-Prolabo, 98.5%), sodium nitrite (Applichem Panreac, 99%), filter paper in reams (Filter Lab, 73 g/m^2^). Azo-bis-isobutyronitrile (AIBN, Aldrich, 98%) was recrystallized twice from methanol.

### 2.2 Instrumentation

Infrared spectra (FTIR) were recorded with an FT/IR-4200 FT-IR Jasco Spectrometer with an ATR-PRO410-S single reflection accessory. High-resolution electron-impact mass spectrometry (EI-HRMS) was performed on a Micromass AutoSpec Waters mass spectrometer (ionization energy = 70 eV; mass resolving power > 10 000). ^1^H and ^13^C{^1^H} NMR spectra were recorded with a Bruker Avance III HD spectrometer operating at 300 MHz for ^1^H, and 75 MHz for ^13^C, using deuterated solvents such as dimethyl sulfoxide (DMSO-*d*_6_) or deuterated chloroform (CDCl_3_) at 25 °C.

Materials were also characterized in terms of their thermal and mechanical properties. Thermogravimetric analysis (TGA) was performed on 10–15 mg of sample under synthetic air and nitrogen atmosphere with a TA Instruments Q50 TGA analyzer, setting the heating rate at 10 °C/min. Differential scanning calorimetry (DSC) used 10–15 mg of the sample under a nitrogen atmosphere with a TA Instruments Q200 DSC analyzer, 20 °C/min. Tensile tests were performed on 5 mm × 9.44 mm × 0.10 mm samples, using a Shimadzu EZ Test Compact Table-Top Universal Tester, setting the rate of separation of jaws at 5 mm/min.

Powder X-ray diffraction (PXRD) patterns were obtained using a Bruker D8 Discover (Davinci design) diffractometer operating at 40 kV, using Cu Kα as the radiation source, a scan step size of 0.02°, and a scan step time of 2 s.

### 2.3 Choice of Main Monomers and Synthesis of Sensory Monomers

These types of chromogenic copolymers are composed of two parts: the sensory motifs and the main monomers. The latter does not interact directly with the target species but has a fundamental role in attaining selective solubility, adjusting the hydrophilic/hydrophobic balance, and preserving or even improving the mechanical properties.^24^ In section SI–S1 of the Supporting Information, we show the initial tests working with several combinations of commercially available main monomers, including, for instance, hydroxyethyl methacrylate and (2-dimethylamino)ethyl methacrylate. These assays resulted in the choice of VP and MMA. Polymers based on these main monomers in equimolar proportions grant a correct fixation onto paper, and that cannot be eluted by water.

On the other hand, the different sensory monomers (MCNs and MCARs) were synthesized from commercially available compounds and with common laboratory equipment. Their synthesis routes and characterization are depicted in section SI–S2 in the Supporting Information.

### 2.4 Polymerization

**Scheme 1:**
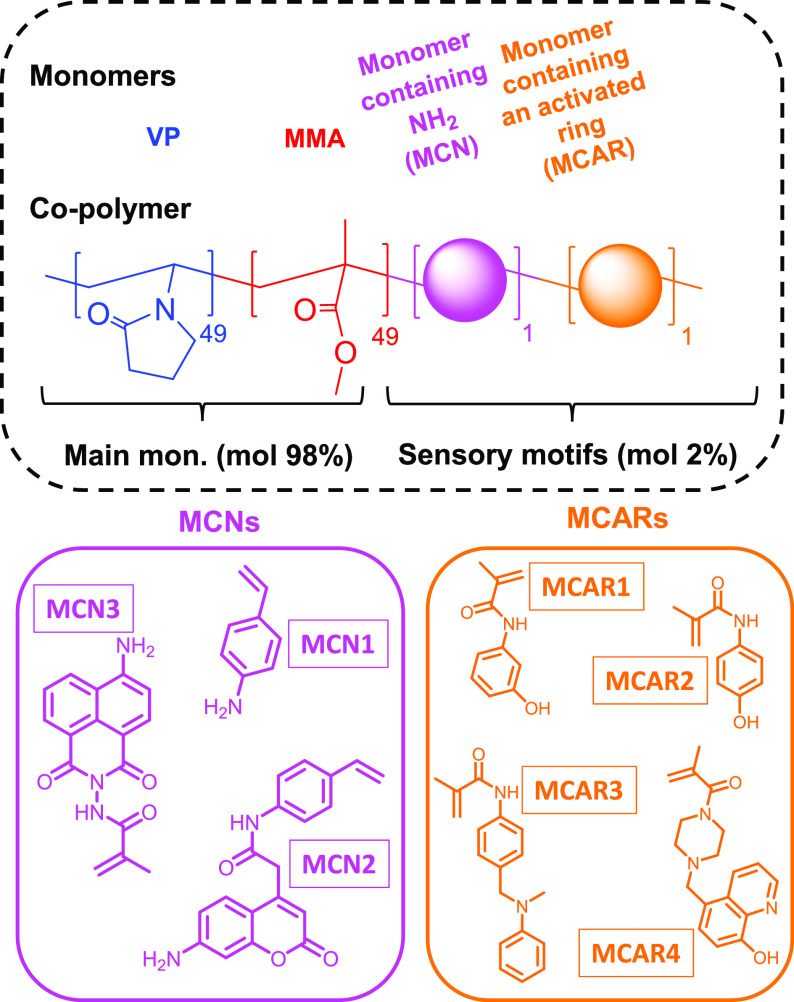
General Structure of Synthesized Polymers, Based on Two Main Monomers and Two Sensory Monomers The copolymer is mainly composed of 1-vinyl-2-pyrrolidone (49 mol %) and methyl methacrylate (49 mol %). The sensory or chromogenic part is based on a monomer containing an amino group (MCN; 1 mol %) and a monomer containing an activated ring (MCAR; mol 1%).

All the synthesized polymers have the same general structure, which is shown in Scheme 1. They were prepared by radical polymerization of the hydrophilic monomer VP, the hydrophobic monomer MMA, MCN, and MCAR in a 49/49/1/1 molar ratio, respectively.

45 mmol of VP, 45 mmol of MMA, 0.94 mmol of MCN, and 0.94 mmol of MCAR were dissolved in DMF (46 mL), and the solution was added to a round-bottom pressure flask. Subsequently, radical thermal initiator AIBN (754 mg, 4.6 mmol) was added, and the solution was sonicated for 10 min; then, it was heated at 60 °C overnight, under a nitrogen atmosphere, and without stirring. After that, the solution was cooled down and dropwise added to diethyl ether (300 mL) with vigorous stirring. All polymers were purified in a Soxhlet apparatus with diethyl ether as the washing solvent.

### 2.5 Paper Coating and Verification

100 mg of sensory polymer were dissolved in acetonitrile (1 mL). Then, 50 μL of the resulting solution were deposited on the surface of a filter paper disk (2 cm diameter, 3.14 cm^2^), and the solvent was evaporated at 60 °C for 5 min.

In a preliminary titration, the synthesized chromogenic polymers were impregnated with aqueous HCl (3.5% w/w) containing sequential additions of NaNO_2_, to define the minimum concentration of this salt. Once a proper concentration was determined on the basis of titration curves, the same acid solution of NaNO_2_ was sprayed over all polymer-coated papers. Then, an aqueous solution of NaOH (1 M) was also pulverized over the disks, immediately revealing an evident color change.

All colors were characterized both by UV–vis spectrophotometry and with a smartphone. For the former, we used an optic fiber accessory for recoding the absorbance spectra. For the latter, more oriented to real use, an iPhone 8 was used for taking a digital photograph and analyzing the RGB parameters (8 bits per channel) with the app “*Colorimetric Titration*”.^25^

## 3 Results and Discussion

### 3.1 Quality and Success of Synthesis

The relatively high amount of AIBN resulted in low-molecular-mass polymers, which favors solubility and does not increase viscosity excessively. The yield, in all cases, was between 80% and 85%.

**Figure 1:**
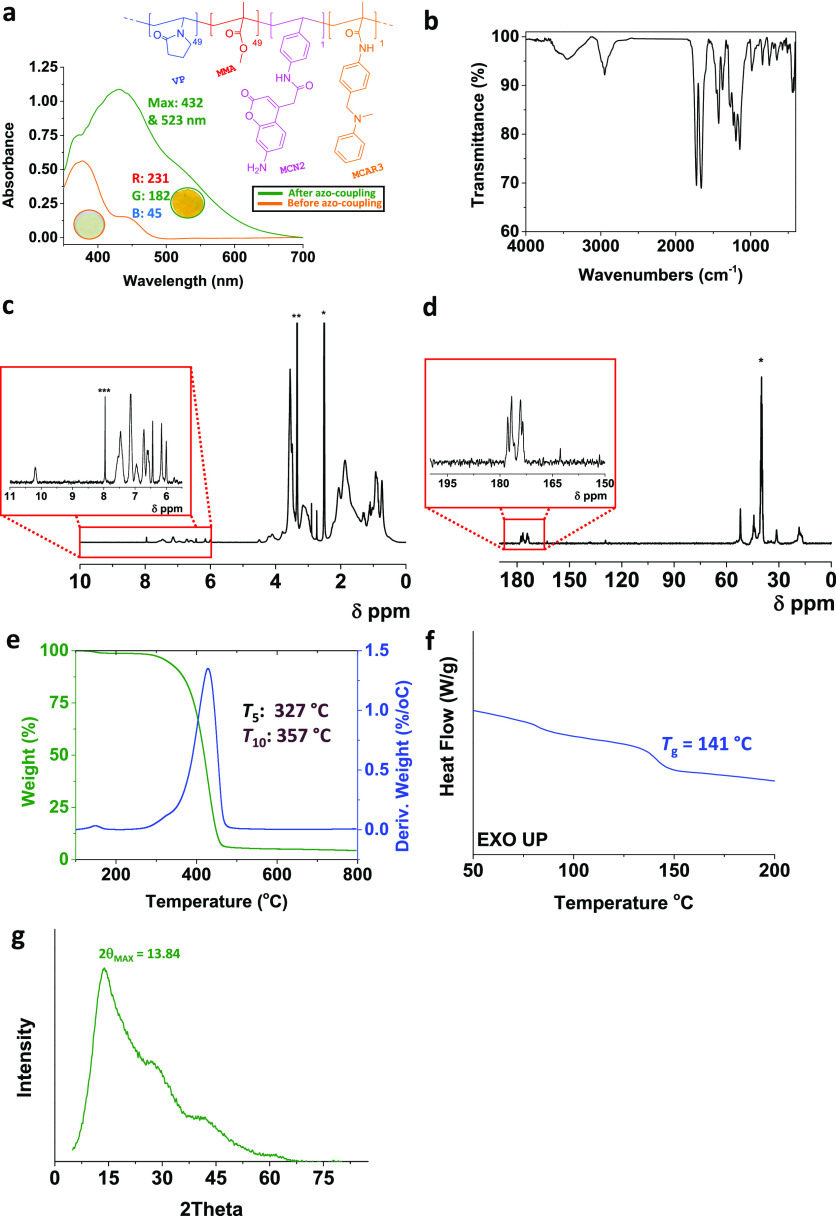
Characterization of polymer 7 (golden) by (a) UV–vis spectrometry, including polymer’s formula and real photographs of coated disks; (b) FT-IR spectroscopy; (c) ^1^H RMN (* = DMSO-*d*_6_, ** = H_2_O). (d) ^13^C RMN (* = DMSO-*d*_6_); (e) thermogravimetric curve at 10 °C min^–1^ under nitrogen atmosphere, showing temperatures *T*_5_ and *T*_10_; (f) DSC curve at a heating rate of 20 °C min^–1^ under a nitrogen atmosphere, showing *T*_g_ value. (g) PXRD spectra showing 2θ_MAX_.

In order to avoid prolixity, the complete characterization of the 12 chromogenic polymers (prepared by combining 3 MCNs and 4 MCARs) can be found in section SI–S3 in the Supporting Information. Figure 1 shows an example for one of the polymers, VP:MMA:MCN2:MCAR3 (polymer number 7). After the azo coupling reaction, its strong electronic absorption across the blue and green regions of the visible light spectrum (Figure 1a) matches its golden color.

The FTIR spectrum (Figure 1b) displays some bands that are common to all polymers, since they are due to MMA and VP, such as the ones at 1623 cm^–1^ (VP) and 1726 cm^–1^ (MMA). But, in addition, the stretching vibrations of C–C bonds in aromatic rings are also evident at ∼1500 cm^–1^. Most of the signals that are due to the N–H and C–N bonds of sensory monomers are overlapped with that of the main monomers, but the strong bands attributed to N–H wag in the lowest energy region (<900 cm^–1^) allow us to distinguish one combination of monomers from another.

For a more accurate distinction, the ^1^H NMR spectrum (Figure 1c) displays at the lowest field a singlet corresponding to the hydrogen of the secondary amide of MCN2 and, while the high field region is dominated by the main monomers, the singlet at 3.0 ppm is not common to all polymers. Indeed, it is due to the methyl group attached to the tertiary amino group of MCAR3. Similarly, the carbonyl carbons of MCN2 are highlighted downfield in the ^13^C NMR spectrum (Figure 1d). In any case, no impurities were detected by NMR spectroscopy.

The thermogram in Figure 1e evidence that the material is not stable beyond 327 °C, and the glass-transition temperature (*T*_g_) of polymer 7 was found to be 141 °C, as shown in Figure 1f. We found similar values for the other polymers (section SI–S3 in the Supporting Information): *T*_5_ ranges from 270 °C to 339 °C and *T*_g_ ranges from 132 °C to 153 °C. These values suggest that these polymers show thermal stability for practical purposes.

In short, monomer synthesis, polymerization, and purification were proved to be successful. For a more extensive characterization, including X-ray diffraction patterns, the reader is referred to section SI–S3.

### 3.2 Verification Methodology: Color Changes

The color change in the papers is based on the reaction between two sensory monomers (MCN and MCAR) in the presence of nitrite anions. Specifically, nitrite anions react with hydrochloric acid in aqueous media, forming nitrosyl anions. This highly reactive chemical species reacts with MCNs, resulting in the formation of a benzene diazonium salt. Finally, this compound attacks the ring’s activated position of MCARs, forming the azo dye (see Figure 4b, presented later in this work).

**Figure 2:**
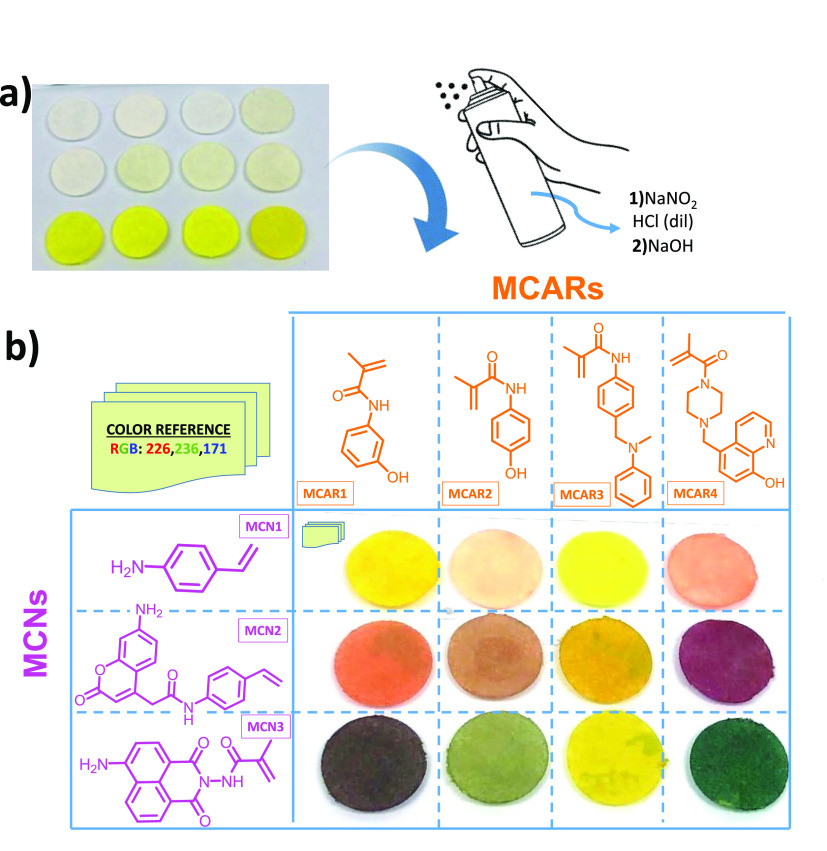
Printed and revealed paper disks: (a) sensory paper disks; (b) revealed disks upon spraying an acid solution of sodium nitrite (0.1 g NaNO_2_, 25 mL water, 2.5 mL HCl (37%)) and an aqueous solution of NaOH (1 M). The matrix shows the 12 different polymers obtained upon combinations of sensory monomers (MCARs and MCNs, 2% mol, each 1%), which were copolymerized with VP (49% mol) and MMA (mol 49%).

Revealing the printed discs involves their spraying with an aqueous acid solution of sodium nitrite (25 mL water, 2.5 mL of concentrated HCl, and 100 mg NaNO_2_), followed by the spraying of an aqueous NaOH 1 M solution for an instantaneous color change (see Figure 2a, and the video provided as Supporting Information). Depending on the chromogenic monomers of choice (MCN and MCAR), different colors were obtained, as shown in Figure 2b.

**Figure 3:**
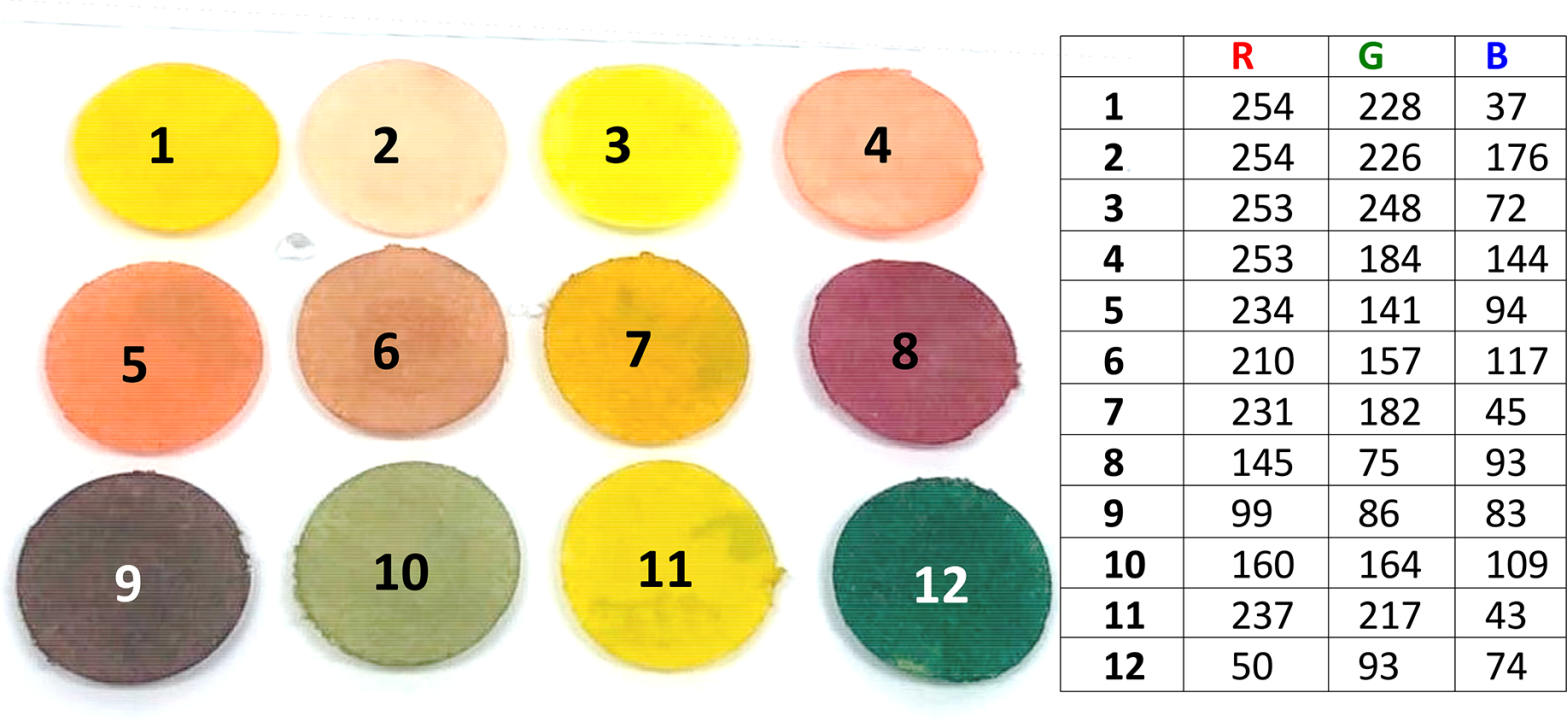
(Left) Filter paper disks coated with sensory polymers after spraying an acid solution of sodium nitrite (0.1 g NaNO_2_, 25 mL water, 2.5 mL HCl (37%)) and an aqueous solution of NaOH (1 M). (Right) RGB color definition parameters for each disk.

Different colors were characterized both by UV–vis spectrophotometry and with a smartphone. For the former, we used an optic fiber accessory for recoding the absorbance spectra. For the latter, more oriented to real use, an iPhone 8 was used to take the digital photo and analyze the RGB parameters with the app “*Colorimetric Titration*”.^25^ In this case, a small piece of plastic was included in the photo as a color reference (see Figure 2b). This element is essential to perform the verification in any lighting condition, since it acts as a reference for adjusting digital color curves, and therefore will always be able to obtain the depicted color definition RGB parameters, as presumed in Figure 3. Furthermore, these color codes are unique for each polymer, so the verification system that we propose is a very powerful, low-cost, and easy-to-use tool. Two examples of real hypothetic applications using 4 of the 12 polymers are shown in the Graphical Abstract, but as many as needed could be used (from 1 to 12), in different positions, different orders, combining various MCNs and MCARs in the same polymer, etc. (see the Video in the Supporting Information).

### 3.3 Study of the Minimum Required Amount of Nitrite

From the chemical point of view, this verification system is basically a nitrite anion sensory system. However, related to the application that we propose for these chromogenic polymers, the typically reported properties for sensors, such as the limits of detection and quantification, are trivial. The lower the nitrite concentration of the spray solution, the lower the intensity of the color formed, which is counterproductive for easy authentication.

**Figure 4:**
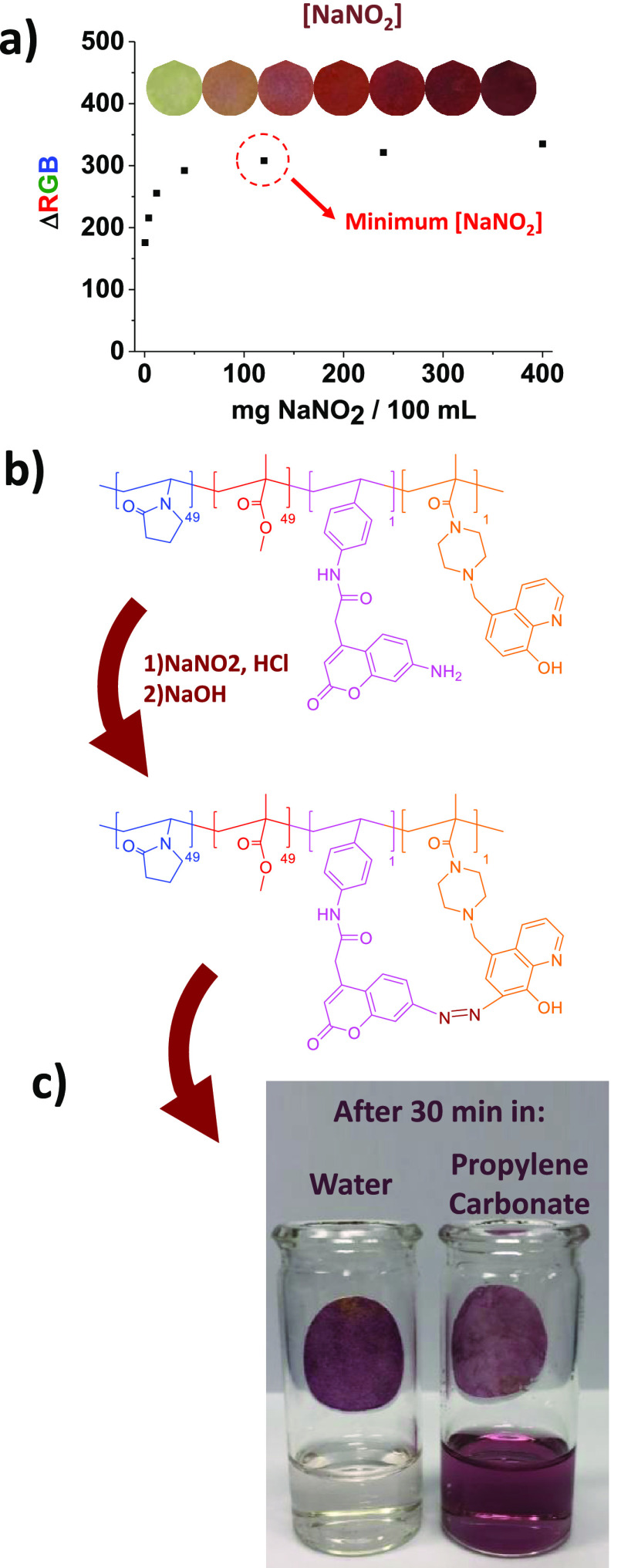
(a) Titration of printed paper disks coated with the sensory polymer VP:MMA:MCN2:MCAR4 (molar ratio of 49:49:1:1, respectively), with NaNO_2_. Each 2-cm-diameter disk was coated with 50 μL of the sensory polymer solution in acetonitrile (100 mg in 1 mL). The coated paper disks were dipped in different NaNO_2_ acid solutions (0.4/4/12/40/120/240 and 400 mg NaNO_2_/100 mL) and finally dipped in aqueous NaOH (1 M). RGB parameters were extracted from the digital photograph, grouped as the unique variable ΔRGB,^26^ and represented against the concentration of NaNO_2_. (b) Azo-coupling reaction in the chromogenic polymer.^21,22^ (c) Colored paper disks after dipping in water and propylene carbonate for 30 min. The picture shows the water-resistant character of the polymer and the solubility in propylene carbonate.

Nonetheless, there will no longer be an increase in color above a specific concentration since all the sensory motifs will have reacted. For this reason, it is important to define the minimum concentration of nitrites in the spray solution, and below which, the colors could be different and not match the RGB code table depicted in Figure 3. Thus, we performed a titration of all chromogenic polymers with sodium nitrite, and we found 120 mg NaNO_2_/100 mL (17.4 mM) as the minimum concentration for an effective spray solution. Figure 4a shows one of the titration curves with one of the polymers as an example (VP:MMA:MCN2:MCAR4 in a 49:49:1:1 molar ratio, respectively), but equivalent results were obtained for the rest of the sensory polymers.

### 3.4 Chemical Recycling; Separation of the Coating and the Paper

As we show in Figure 4c, the coating can be separated from the paper in a very simple way, simply by treating the paper with a green solvent such as propylene carbonate. In this way, and despite having a minimum amount of polymer (1.6 mg of polymer per cm^2^ of paper), our sensory polymer does not imply any paper recycling problem. The copolymers have been specifically designed to be water-resistant for practical applicability of the intelligent labels, but, at the same time, are easily removable with the appropriate green solvent.

### 3.5 Study of the Effect of the Coating on Paper’s Mechanical Properties

The coating of papers (or specific paper areas) in no case can suppose the loss of properties, which is reason why a comparative study was performed analyzing the Young’s modulus of the paper without and with coating. Since 98% of the polymers’ chemical composition is identical in the 12 examples (49 mol % VP and 49 mol % MMA), this study was only performed with the polymer VP:MMA:MCN2:MCAR4 (molar ratio 49:49:1:1, respectively). The Young’s modulus for the paper with and without the coating was 491 and 511 MPa, respectively. Thus, we did not find significant differences between them.

### 3.6 Proof of Concept. Encrypting and Decrypting

We are describing a cryptographic system for securing the authenticity of a branded product. This system is partially inspired by the asymmetric public-key/private-key cryptography that secures electronic communications,^22^ and we believe a real example would be clarifying for the reader.

In this example, the dispatcher of a branded watch devises a color code, which may be different for every single product, and keeps it private. For instance, we may suggest a simple four-color code: R234/G141/B94 (salmon) – R59/G93/B74 (dark sea green) – R145/G75/B93 (purple) – R231/G182/B45 (golden). This code is then encrypted as an apparently dull label of white/colorless and yellow elements (public key). Encrypting is accomplished by printing or coating up to 12 chromogenic polymers on the paper label, if not on the packaging paper directly, knowing in advance how each of those polymers looks before and after applying a verification solution. We can easily see that the series of polymers required in this example is 5 – (MCN2, MCAR 1) – 12 (MCN3, MCAR4) – 8 (MCN2, MCAR4) – 7 (MCN2, MCAR3).

When customers receive the package containing the watch, they can decrypt the code by applying the revealing solution with a 2-fold aim: guaranteeing that the key has not been previously cracked and checking that the code matches the one provided by the manufacturer. Moreover, the dispatcher can send the unique color code electronically only at the moment the purchaser wants, increasing the security of the verification system.

The number of combinations is dependent on the number of polymers, e.g., if the 12 polymers are printed, their permutation without repetition, where the linear order is relevant, gives 12! possibilities (*V*_*k*_(*n*), where *n* and *k* = 12), broadly 479 million of possibilities. Moreover, if the 12 polymers are printed with repetition, the possibilities are n^12^ (*V’*_*k*_(*n*), where *n* and *k* = 12), broadly 9 trillion possibilities.

## 4 Conclusions

We have designed an anticounterfeiting system based on 12 chromogenic polymers applied directly to the paper as coatings. The intelligent colorless label is printed on the paper as an array of printed disks or squares of these polymers in a combination that allows for trillions of possibilities if, for example, the array comprised 12 disks, including repetition of the polymers. The intelligent labels are cryptographic keys belonging to the owner that can be revealed into a color chart for checking the inviolability of the key and the coincidence of the owner key with the revealed label. From a practical viewpoint, the label changes into the color chart of the owner’s cryptographic key when sprayed using a solution of sodium nitrite in acid medium, followed by a solution of NaOH (1 M). This expectation, i.e., that the end-user will handle moderately acidic solutions and alkalis with due safety, is the only drawback of this approach, and hopefully it will be overcome by using impregnated foam rollers or stamps, which could be easily commercialized.

The color change of each sensory polymer is depends on the sensory monomers used in the polymer synthesis, and, as mentioned, we have made 12 combinations of chromogenic monomers that generate 12 different colors. For more accurate identification of these colors, the user can quantify the RGB coordinates against a certain reference and compare them with the ones provided by the dispatcher. The integration of the sensory polymers in the paper does not significantly modify the mechanical properties of the paper. While when discrete molecules are used in solution for paper coating, the chemical species are easily eluted from the substrate, the incorporation of the sensory motifs as comonomers of a VP/MMA-based polymer grants both water resistance and nonmigration. At the same time, polymers have been designed to be soluble in the appropriate solvent, so that it is easily removable from the paper for appropriate recycling of both parts.

## Supporting Information Available

Selection of the best main monomers; synthesis and characterization of MCNs and MCARs; characterization of sensory polymers (PDF)
Proof of concept video (AVI)

## Supplementary Material

am1c19228_si_001.pdf

am1c19228_si_002.avi
