# Supplementary material for: Chromogenic Anticounterfeit and Security Papers: An Easy and Effective Approach
Source: ACS Appl Mater Interfaces. 2021 Dec 7;13(50):60454–61. doi: 10.1021/acsami.1c19228 (PMC8704190; doi:10.1021/acsami.1c19228)
Supplement: Supplementary file 1 — am1c19228_si_001.pdf [file am1c19228_si_001.pdf]

Supporting Information (SI)

CHROMOGENIC ANTI-COUNTERFEIT AND SECURITY PAPERS: AN EASY AND EFFECTIVE APPROACH

José Carlos GUIRADO-MORENO[a], Marta GUEMBE-GARCÍA[a], José M. GARCÍA[a], Roberto AGUADO,[b] Artur J.M. VALENTE,[b]*Saúl VALLEJOS[a,b,]*

[a] Departamento de Química, Facultad de Ciencias, Universidad de Burgos, Plaza de Misael Bañuelos s/n, 09001 Burgos, Spain

[b] University of Coimbra, CQC, Department of Chemistry, Rua Larga, 3004-535 Coimbra, Portugal

*Corresponding authors: Dr Valente (avalente@ci.uc.pt); Dr Vallejos (svallejos@ubu.es)

Table of contents

**S1.** Selection of the best main monomers	2
**S2.** Synthesis and characterization of MCNs and MCARs	3
**S3.** Characterization of sensory polymers	16

S1. Selection of the best main monomers

Preliminary study with different main monomers was carried out by fixing MCN1 and MCAR1 as the sensory monomers, and varying the commercially available monomers.

The aim of these tests was to find the best combination of main monomers in terms of color homogeneity and water resistance resistance, i.e., absence of migration of color upon contacting the paper with water. As shown in **Figure S1**, 4 different combinations of main monomers were synthesized and tested. In some of them, the development of color in the paper discs was not homogeneous, indicating that the coating or printingof the polymer on the paper fibers was not uniform. Second, other combinations of main monomers were found that did present a homogeneous dispersion on the paper, but after spraying the acidic nitrite and NaOH solutions the polymer was dissolved in water, as shown like a residue in **Figure S1**. For the selected application, we chose VP and MMA (number 4) as the best combination of main monomers, because no migration and an homogenus change of color was observed.

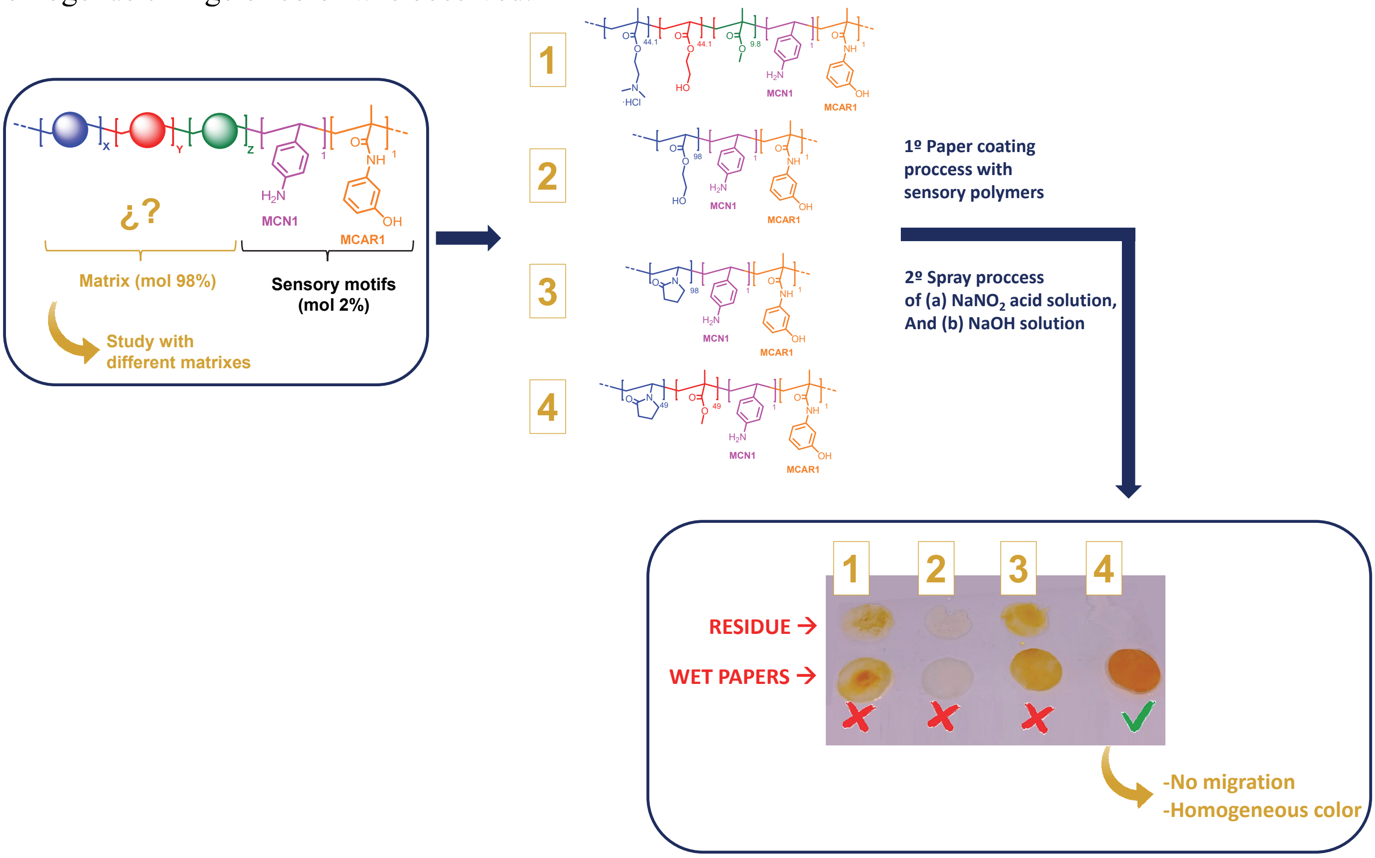

Figure S1. Preliminary study with different main monomers, and fixed sensory monomers (MCN1 and MCAR1). After spraying sodium nitrite and sodium hydroxide solutions, the only polymer which showed water resistance (no migration and water insolubility) and a homogeneous color change was polymer number 4, made of 1-vinyl-2-pyrrolidone and methyl methacrylate.

S2. Synthesis and characterization of MCNs and MCARs

Monomers containing NH_2 group (MCNs)

MCN1

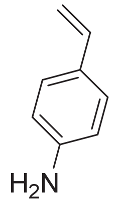

MCN1
CAS: 1520-21-4

MCN2

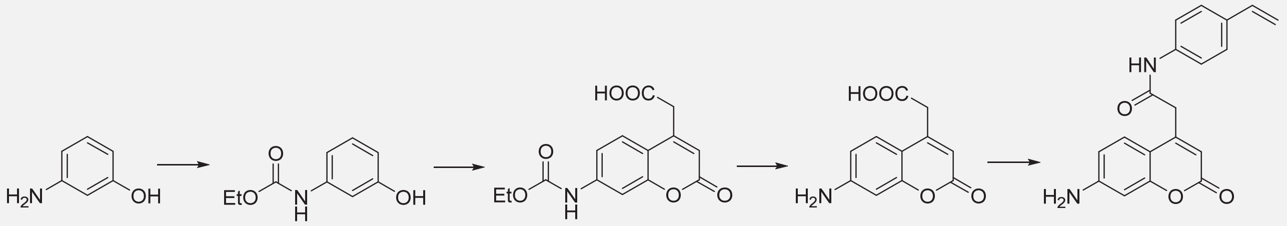

CAS: 591-27-5 | 1 | 2 | 3 | MCN2

MCN3

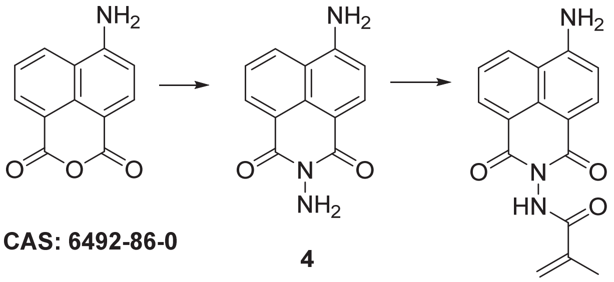

CAS: 6492-86-0 | 4 | MCN3

Scheme S1. Followed synthetic routes for different MCNs.

Monomers containing activated rings (MCARs)

MCAR1

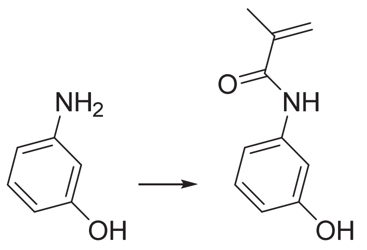

CAS: 591-27-5 MCAR1

MCAR2

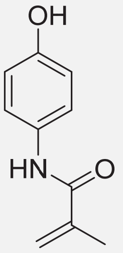

MCAR2
CAS: 19243-95-9

MCAR3

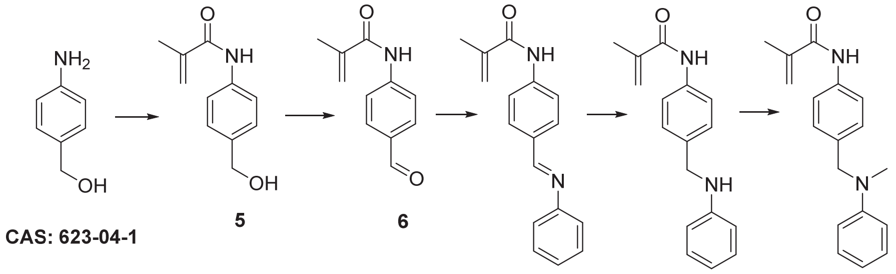

7 8 MCAR3

MCAR4

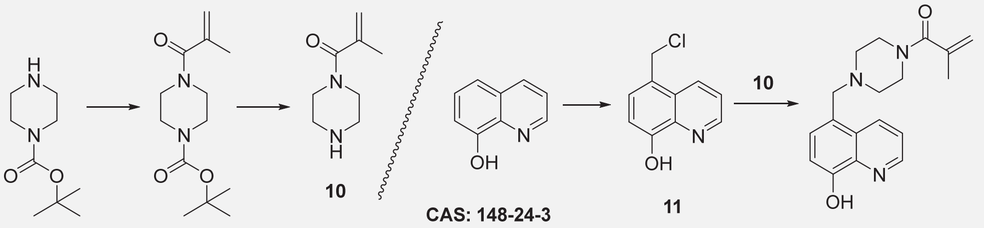

CAS: 57260-71-6 9 MCAR4

Scheme S2. Followed synthetic routes for different MCNs.

- ***Synthesis of ethyl (3-hydroxyphenyl)carbamate (1)***

Synthesis and characterization carried out as described by *Kellner et al.*[1]

- ***Synthesis of 2-(7-((ethoxycarbonyl)amino)-2-oxo-2H-chromen-4-yl)acetic acid (2)***

Synthesis and characterization carried out as described by *Thorson et al.*[2]

- ***Synthesis of 2-(7-amino-2-oxo-2H-chromen-4-yl)acetic acid (3)***

Synthesis and characterization carried out as described by *Thorson et al.*[2]

- ***Synthesis of 2-(7-amino-2-oxo-2H-chromen-4-yl)-N-(4-vinylphenyl)acetamide (MCN2)***

A mixture of 2-(7-amino-2-oxo-2H-chromen-4-yl)acetic acid (3) (3.94 g, 17,98 mmol), N,N'-diciclohexilcarbodiimide (3.98 g, 18.85 mmol), 4-vinylaniline (2.1 mL, 2.14 g, 17.98 mmol) and 200 mL of THF was stirred overnight in a pressured flask at 45 °C. After that, the mixture was filtered and the solvent was removed under reduced pressure. Finally, the solid was washed with 100mL of DCM. Yield: 50%.^{1}H NMR (300 MHz, DMSO-d_6) δ (ppm) = 10.34 (s, 1H), 7.58 (d, J = 8.6 Hz, 2H), 7.48 (d, J = 15.4, 1H), 7.43 (d, J = 8.6 Hz, 2H)6.67 (dd, J = 17.7, 11.0 Hz, 1H), 6.57 (d, J = 8.7 Hz, 1H), 6.45 (s, 1H), 6.16 (s, 2H), 6.02 (s, 1H), 5.74 (d, J = 17.6 Hz, 1H), 5.18 (d, J = 11.7 Hz, 1H), 3.82 (s, 2H).^{13}C NMR (75 MHz, DMSO-d_6,) δ (ppm) = 167.24, 161.11, 156.14, 153.59, 151.52, 139.02, 136.54, 132.90, 127.08, 126.78, 119.64, 113.39, 111.70, 109.33, 108.75, 99.06. HRMS (EI) *m/z* $[M+H]^+$ calc.: 231.1161; found: 321.1236.

a)

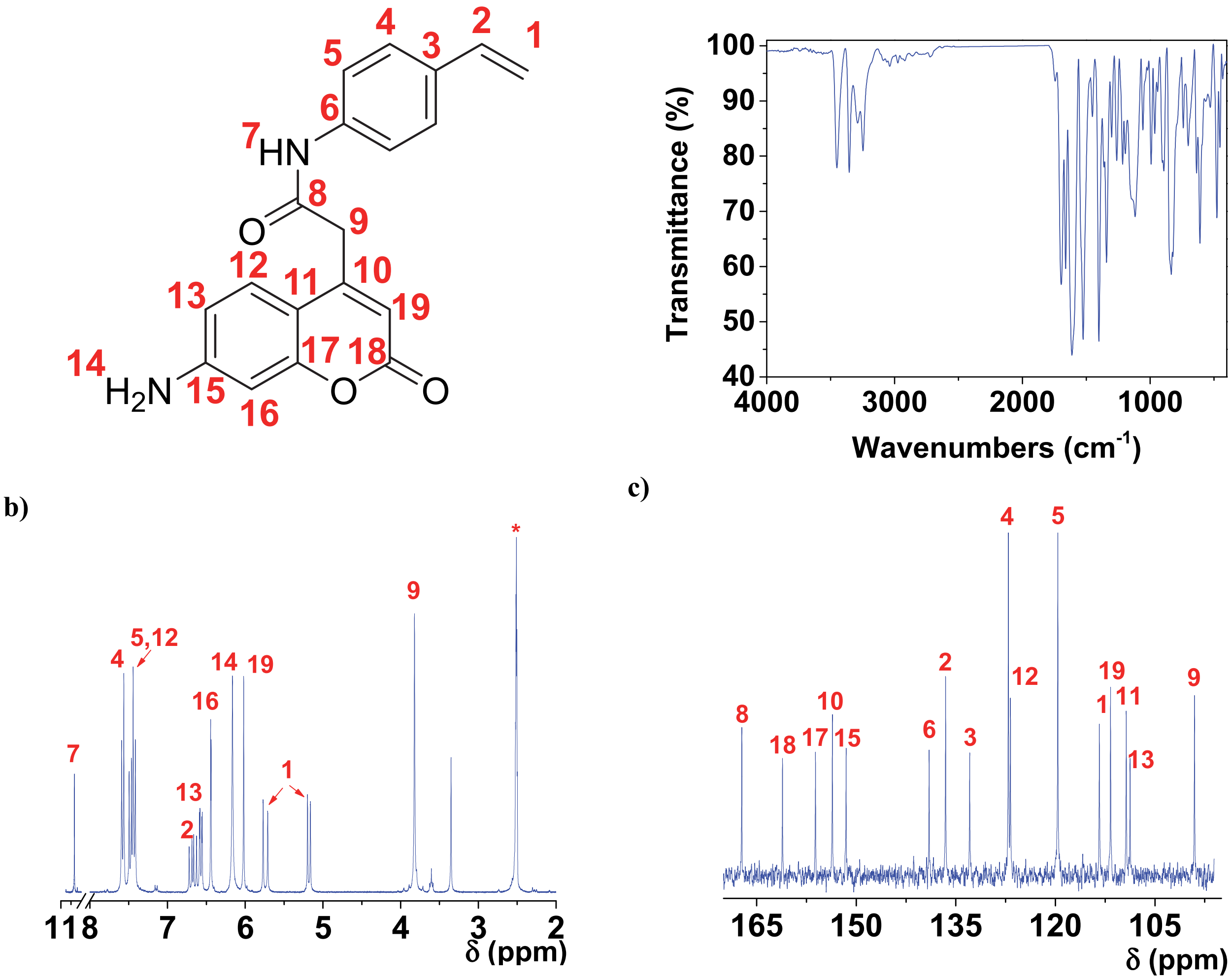

Figure S2. Characterization of MCM**2** by (a) FTIR, (b) ^{1}H NMR, and (c) ^{13}C NMR spectroscopies (* = solvent signal, DMSO-d_6).

- ***Synthesis of 2,6-diamino-1H-benzo[de]isoquinoline-1,3(2H)-dione (4)***

A mixture of 4-amino-1,8-naphtalic anhydride (0.39g, 1.83mmol), hydrazine monohydrate (0.178mL, 3.66mmol) and ethanol (10mL) was stirred at 100ºC for 4 hours. Then, the mixture was cooled, and the solid (0.36g, 1.58mmol) was filtered and washed with ethanol. Yield: 87%. ^{1}H NMR (300 MHz, DMSO-d_6) δ (ppm) = 8.62 (d, J = 6.6 Hz, 1H), 8.42 (d, J = 6.6 Hz, 1H), 8.19 (d, J = 7.5 Hz, 1H), 7.64 (s, 1H), 7.51 (s, 2H), 6.84 (d, J = 7.4 Hz, 1H), 5.73 (s, 2H). ^{13}C NMR (75 MHz, DMSO- d_6) δ (ppm) = 160.55, 153.60, 134.55, 131.41, 129.82, 128.61, 124.44, 121.72, 119.87, 108.74, 107.31. HRMS (EI) *m/z* $[M+H]^+$ calc.: 227.0768; found: 228.0766

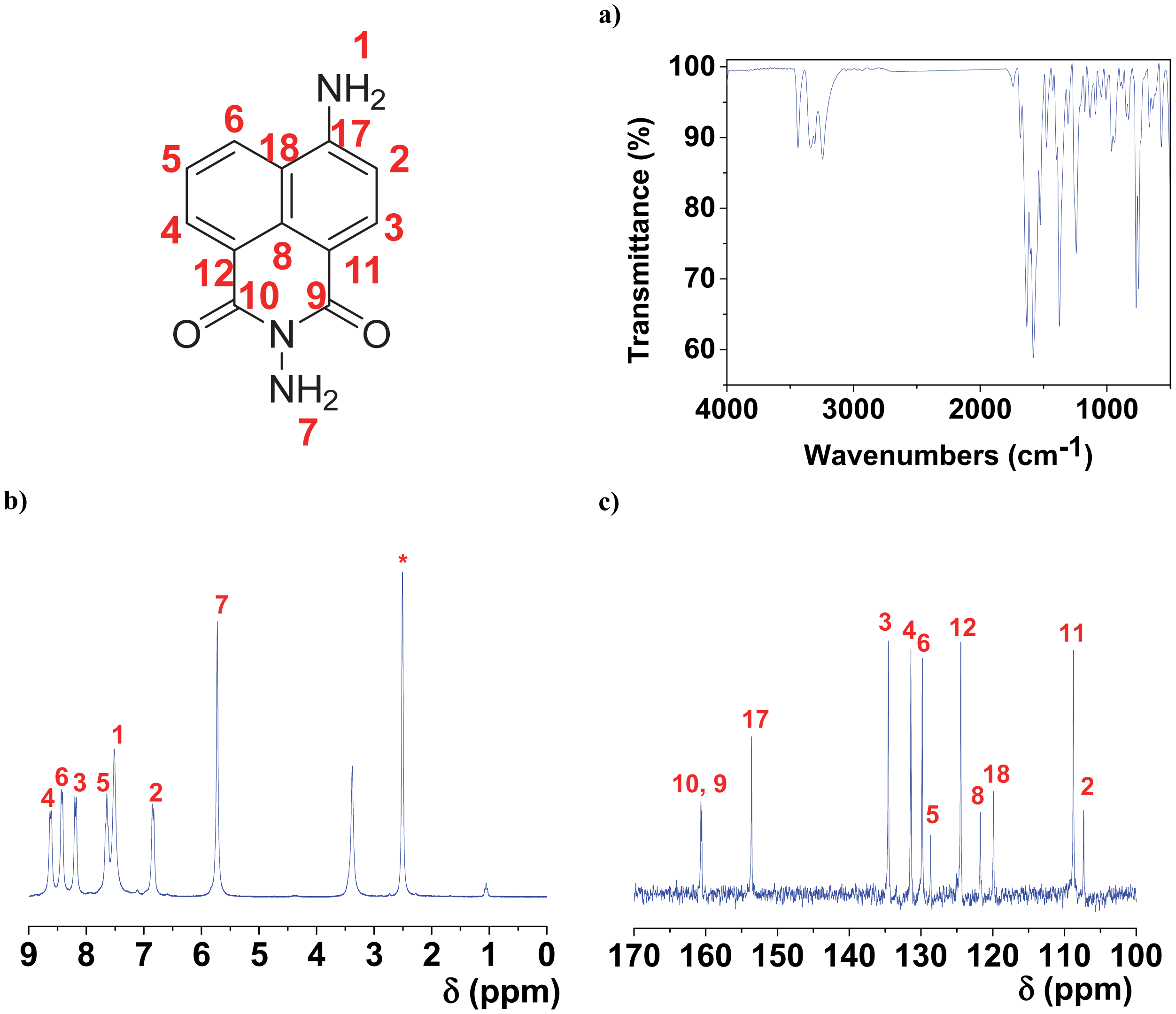

Figure S3. Characterization of MCM**2** by (a) FTIR, (b) ^{1}H NMR, and (c) ^{13}C NMR spectroscopies (* = solvent signal, DMSO-d_6).

- ***Synthesis of N-(6-amino-1,3-dioxo-1H-benzo[de]isoquinolin-2(3H)-yl)methacrylamide (MCN3)***

2,6-diamino-1H-benzo[de]isoquinoline-1,3(2H)-dione **(4)** (0.483g, 2.12mmol) was dissolved in THF, then triethylamine (0.246mL, 2.43mmol) and methacryloyl chloride (0.207mL, 2.12mmol) were added. The mixture was stirred overnight under nitrogen atmosphere at 90ºC. After that, the mixture was cooled and filtered, then the solvent was removed under vacuum to obtain a solid (442 mg, 1.5 mmol), finally it was washed with water. Yield: 71%. ^{1}H NMR (300 MHz, DMSO-d_6) δ (ppm) = 10.56 (s, 1H), 8.68 (d, J = 8.4 Hz, 1H), 8.47 (d, J = 8.1 Hz, 1H), 8.22 (d, J = 8.4 Hz, 1H), 7.77 – 7.65 (m, 1H), 7.59 (s, 2H), 6.89 (d, J = 8.5 Hz, 1H), 5.97 (s, 1H), 5.62 (s, 1H), 1.97 (s, 3H). ^{13}C NMR (75 MHz, DMSO-d_6) δ (ppm) = 166.71, 162.65, 161.66, 153.78, 138.13, 134.96, 132.10, 130.21, 124.61, 122.06, 120.00, 108.91, 107.40, 104,09, 82.27, 18.93. HRMS (EI) m/z $[M+H]^+$ calc.: 296.1035; found: 296.1032

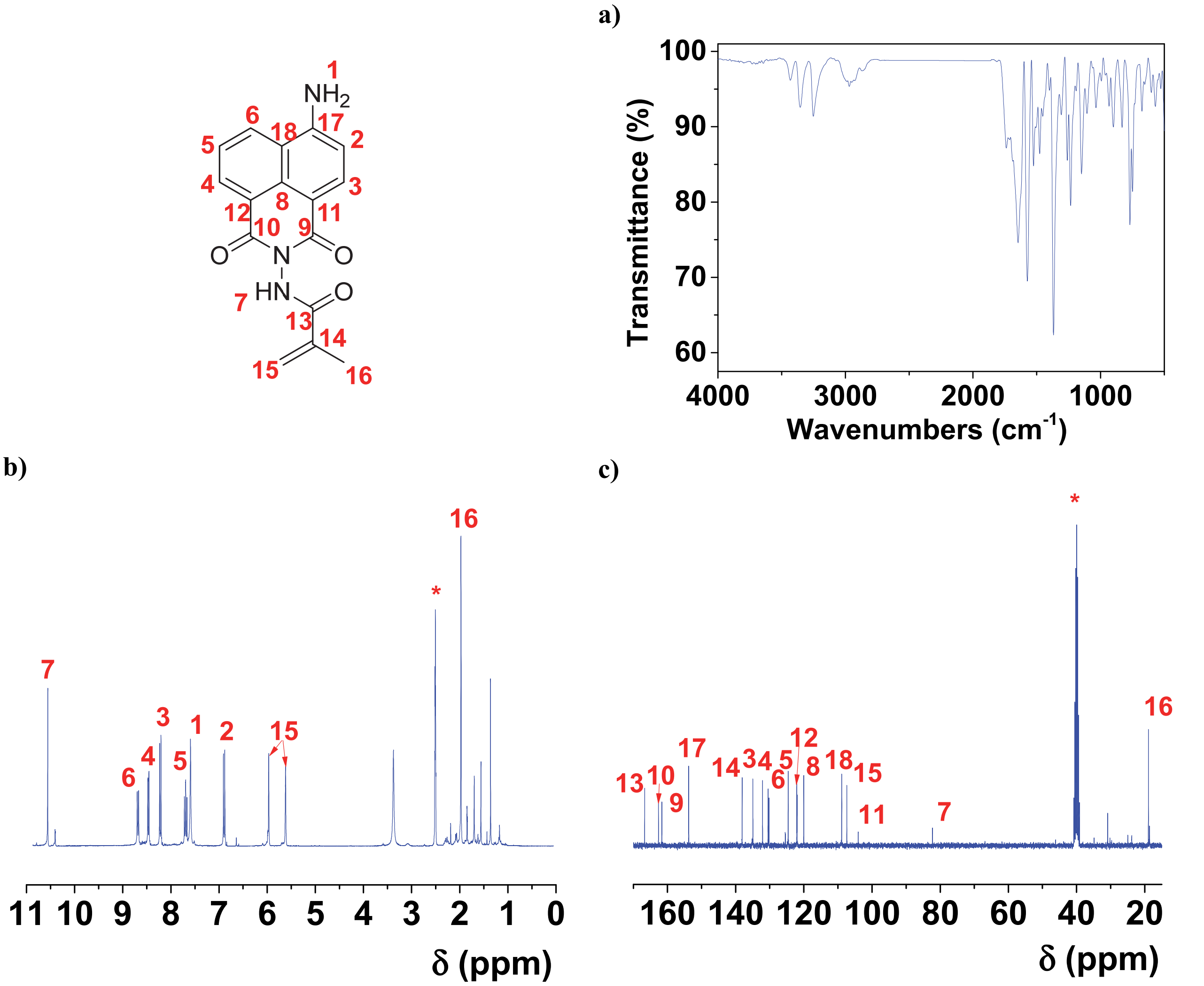

Figure S4. Characterization of MCM**2** by (a) FTIR, (b) ^{1}H NMR, and (c) ^{13}C NMR spectroscopies (* = solvent signal, DMSO-d_6).

- ***Synthesis of N-(3-hydroxyphenyl)methacrylamide (MCAR1)***

Synthesis and characterization carried out as described by *Guembe et al.*[3]

- ***Synthesis of N-(4-(hydroxymethyl)phenyl)methacrylamide (5)***

4-aminophenylmethanol (10.22g, 83mmol) (12.38mL, 83mmol) was dissolved in ethyl acetate (50mL), and methacrylic anhydride was added. The mixture was stirred overnight at room temperature, and the solvent was removed under reduced pressure. Finally, the solid (15.12g, 79.07mmol) was washed with hexane and filtered. Yield: 95%. ^{1}H NMR (300 MHz, DMSO -d_6) δ (ppm) = 9.73 (s, 1H), 7.63 (d, J = 8.6 Hz, 2H), 7.25 (d, J = 8.7 Hz, 2H), 5.79 (s, 1H), 5.48 (s, 1H), 5.10 (t, J = 5.7 Hz, 1H), 4.45 (d, J = 5.5 Hz, 2H), 1.95 (s, 3H). ^{13}C NMR (75 MHz, DMSO -d_6) δ (ppm) = 167.15, 140.93, 138.09, 127.19, 120.46, 120.24, 63.13, 19.23. HRMS (EI) m/z $[M+H]^+$ calc.: 192.1025; found: 192.1021.

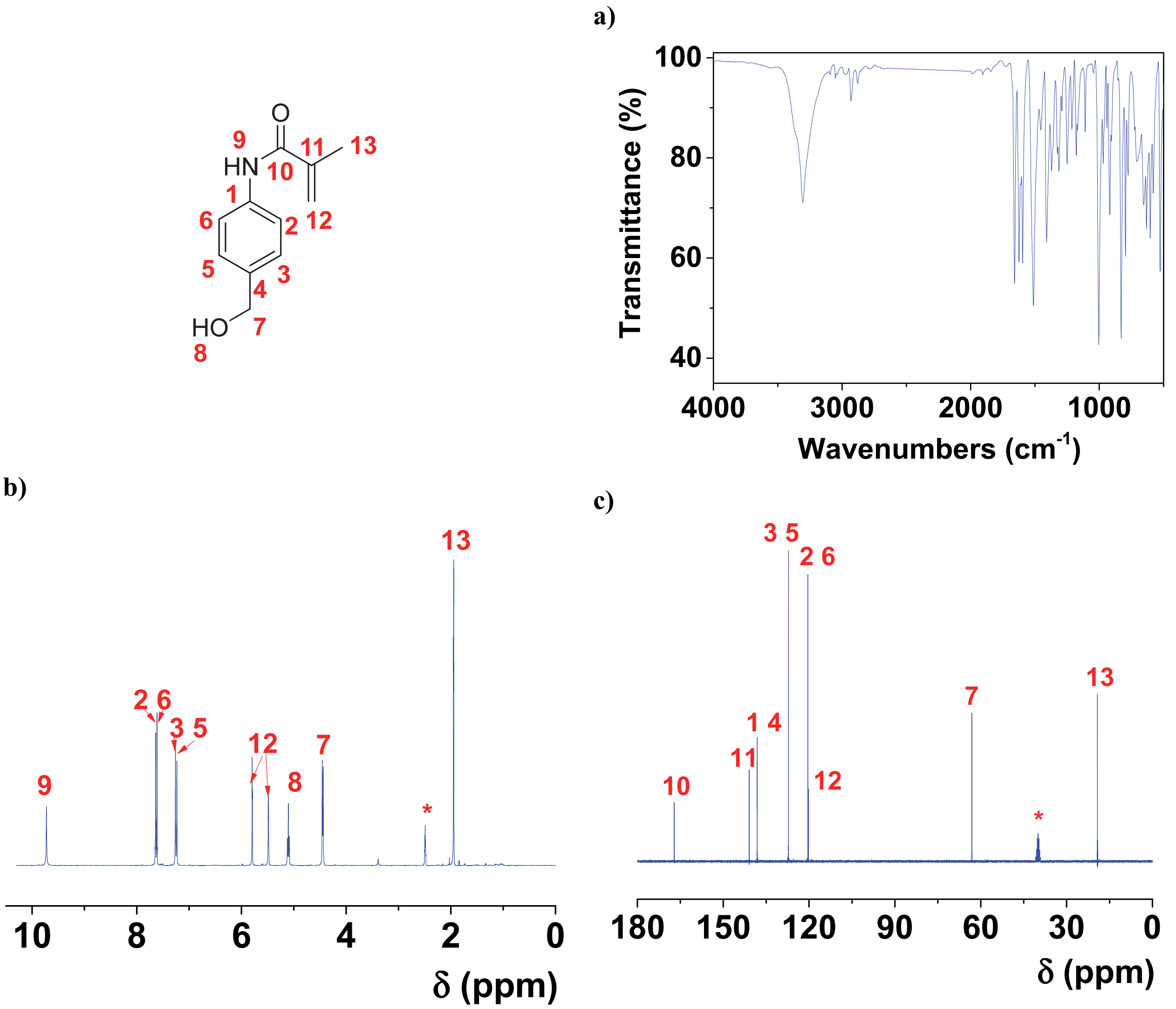

Figure S5. Characterization of MCM**2** by (a) FTIR, (b) ^{1}H NMR, and (c) ^{13}C NMR spectroscopies (* = solvent signal, DMSO-d_6).

- ***Synthesis of N-(4-formylphenyl)methacrylamide (6)***

N-(4-(hidroxymethyl)phenyl)metacrylamide (5) (4,6g, 24.05mmol) was dissolved in DCM (25mL), and manganese oxide (17g, 195mmol) was added. The mixture was stirred overnight at room temperature and then it was filtered with celite and the solvent was removed under reduced pressure. Finally, the white solid (4.01g, 21.19mmol) was filtered and washed with hexane. Yield: 88%. ^{1}H NMR (300 MHz, DMSO -d_6) δ (ppm) = 10.15 (s, 1H), 9.87 (s, 1H), 7.93 (d, J = 8.7 Hz, 2H), 7.84 (d, J = 8.9 Hz, 2H), 5.86 (s, 1H), 5.57 (s, 1H), 1.95 (s, 3H). ^{13}C NMR (75 MHz, DMSO -d_6) δ (ppm) = 191.95, 167.77, 145.20, 140.57, 131.87, 131.00, 121.30, 120.09, 19.06. HRMS (EI) m/z [M+Na]$^+$ calc.:212.0687; found: 212.0682.

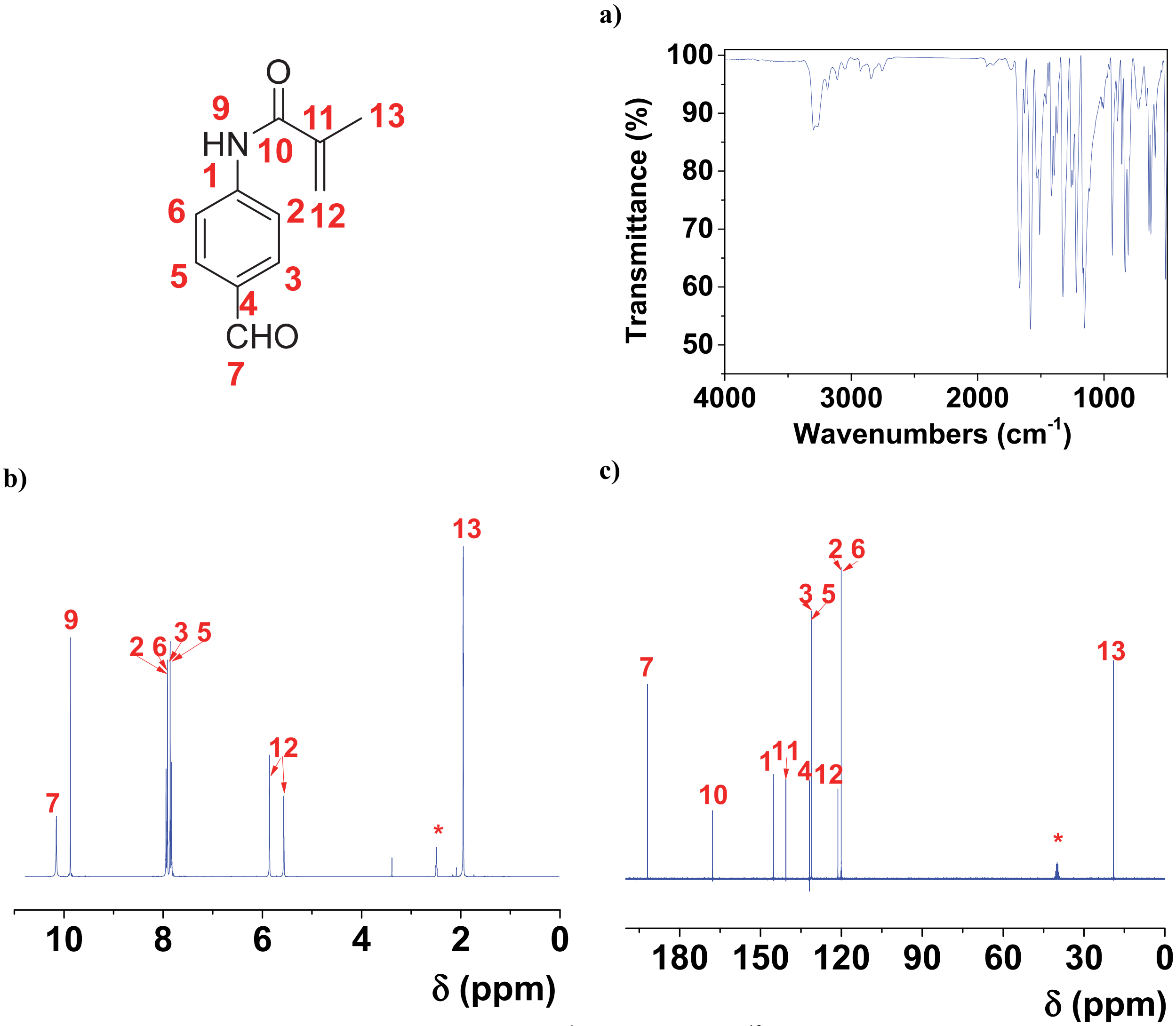

Figure S6. Characterization of MCM**2** by (a) FTIR, (b) ^{1}H NMR, and (c) ^{13}C NMR spectroscopies (* = solvent signal, DMSO-d_6).

- ***Synthesis of (E)-N-(4-((phenylimino)methyl)phenyl)methacrylamide (7)***

A mixture of *N*-(4-(formylphenyl)methacrylamide (6) (2.17g, 11.47mmol), aniline (1.57mL, 17.2mmol) and hexane (250mL) was stirred 3,5 hours at 100ºC. After that, the suspension was filtered and washed with hexane to obtain a white solid (2.59g, 9.80mmol). Yield: 85%. ^{1}H NMR (300 MHz, DMSO -d_6) δ (ppm) = 10.05 (s, 1H), 8.54 (s, 1H), 8.03 – 7.80 (m, 4H), 7.49 – 7.33 (m, 2H), 7.33 – 7.15 (m, 3H), 5.86 (s, 1H), 5.57 (s, 1H), 1.98 (s, 3H). ^{13}C NMR (75 MHz, DMSO -d_6) δ (ppm) = 167.54, 160.38, 152.16, 142.49, 140.71, 131.62, 129.80, 129.65, 126.18, 121.41, 120.94, 120.28, 19.17. HRMS (EI) m/z $[M+H]^+$ calc.: 265.1341; found: 265.1337

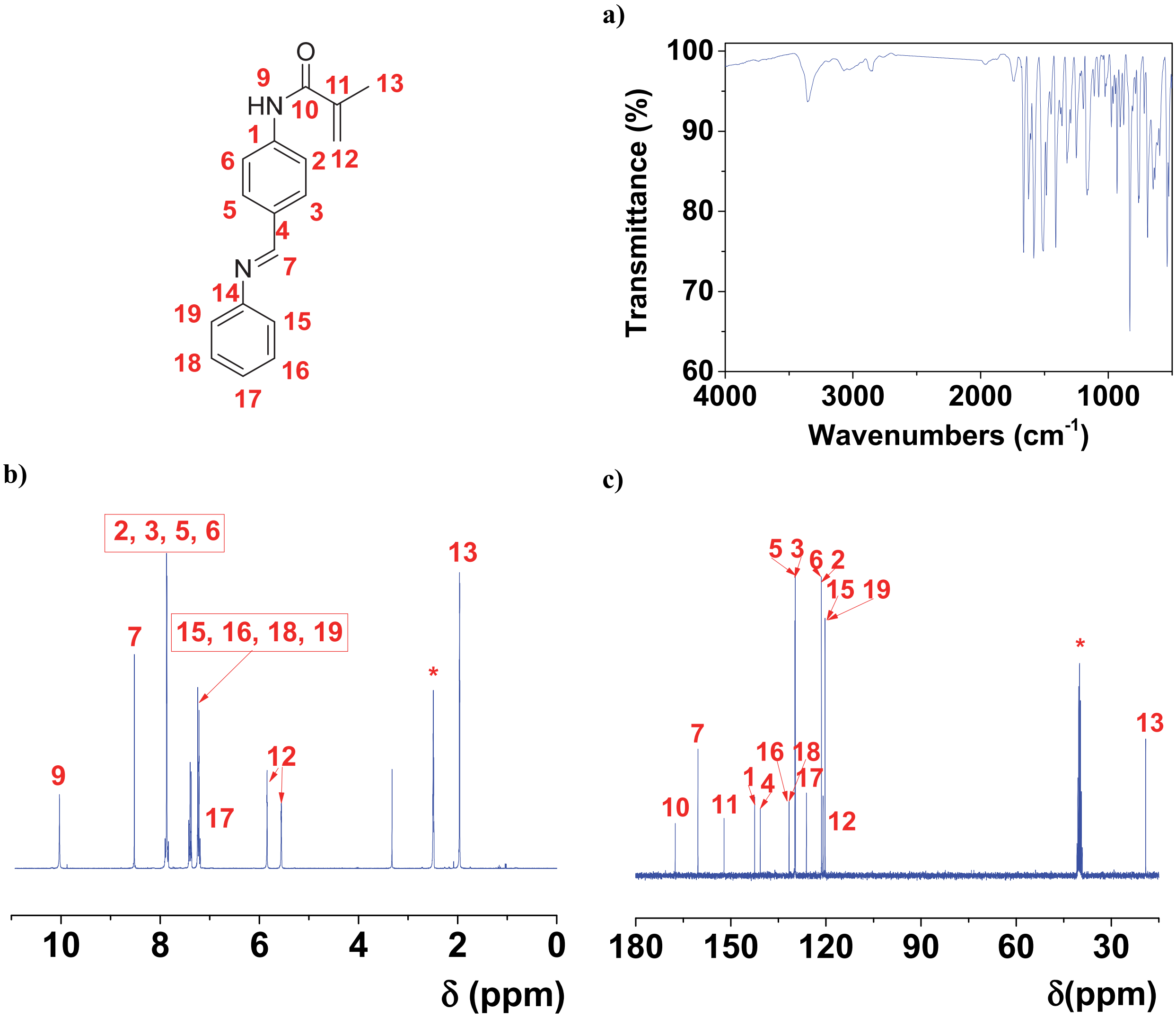

Figure S7. Characterization of MCM**2** by (a) FTIR, (b) ^{1}H NMR, and (c) ^{13}C NMR spectroscopies (* = solvent signal, DMSO-d_6).

- ***Synthesis of N-(4-((phenylamino)methyl)phenyl)methacrylamide (8)***

(E)-N-(4-((phenylimino)methyl)phenyl)methacrylamide (7) (1.5g, 5.67mmol) was dissolved in methanol (75mL), then sodium borohydride (1.037g, 5.16mmol) was slowly added and the mixture was stirred 1 hour at room temperature. After that, the solvent was removed under reduced pressure, finally the white solid (1.37g, 5.16mmol) was was filtered and washed with distilled water. Yield: 91%.^{1}H NMR (300 MHz, DMSO -d_6) δ (ppm) = 7.63 (d, J = 8.5 Hz, 2H), 7.29 (d, J = 8.5 Hz, 2H), 7.10 – 6.93 (m, 2H), 6.57 (d, J = 7.7 Hz, 2H), 6.50 (t, J = 7.3 Hz, 1H), 6.13 (t, J = 5.9 Hz, 1H), 5.80 (s, 1H), 5.49 (s, 1H), 4.22 (s, 2H), 1.95 (s, 3H). ^{13}C NMR (75 MHz, DMSO -d_6) δ (ppm) = 167.16, 149.14, 140.94, 138.07, 135.75, 127.80, 120.71, 120.25, 112.81, 46.64, 19.24. HRMS (EI) m/z [M+H]$^+$ calc.: 267.1497; found: 267.1492

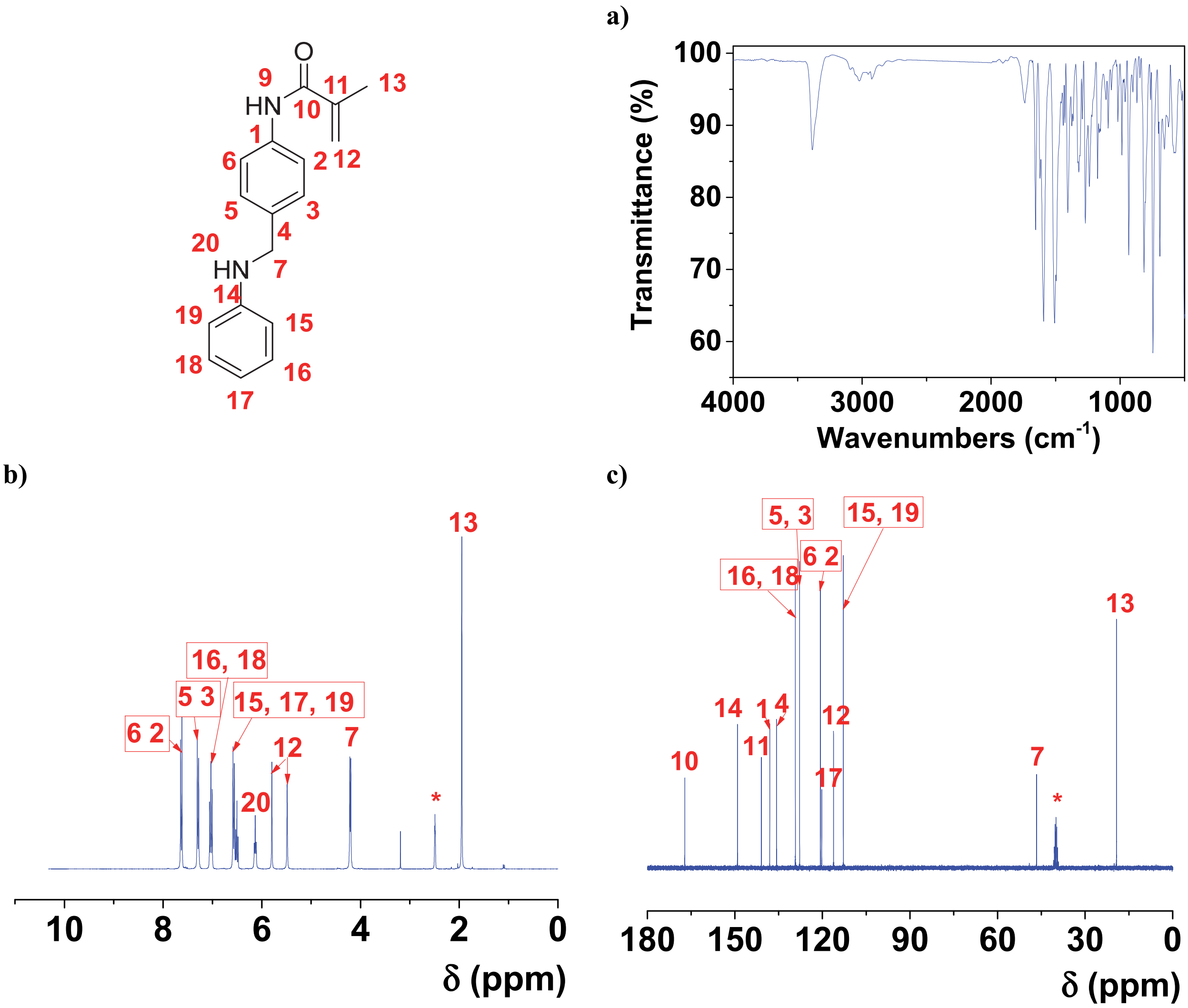

Figure S8 Characterization of MCM**2** by (a) FTIR, (b) ^{1}H NMR, and (c) ^{13}C NMR spectroscopies (* = solvent signal, DMSO-d_6).

- ***Synthesis of N-(4-((methyl(phenyl)amino)methyl)phenyl)methacrylamide (MCAR3)***

To a stirred mixture of N-(4-((phenylamino)methyl)phenyl)methacrylamide **(8)** (1.02g, 3.83mmol), paraformaldehyde (1.248g, 37.48mmol), and sodium borohydride (0.734g, 19.4mmol) in THF (50mL) at room temperature under nitrogen was added dropwise trifuoroacetic acid (18.7mL, 244.18mmol). The resulting mixture was stirred overnight at room temperature, the poured into a mixture of 20% aqueous NaOH (70mL) and ice. After that, the mixture was diluted with brine (70mL), and extracted with DCM (3x70mL). The combined organic layers were dried, and the solvent was removed under vacuum to obtain a white solid (0.860g, 3.07mmol). Yield: 80%. ^{1}H NMR (300 MHz, DMSO -*d*6) δ (ppm) = 9.75 (s, 1H), 7.61 (d, J = 8.6 Hz, 2H), 7.23 – 7.05 (m, 4H), 6.72 (d, J = 7.9 Hz, 2H), 6.61 (t, J = 7.2 Hz, 1H), 5.79 (s, 1H), 5.50 (s, 1H), 4.50 (s, 2H), 2.98 (s, 3H), 1.94 (s, 3H). ^{13}C NMR (75 MHz, DMSO -*d*6) δ (ppm) = 167.15, 149.60, 140.89, 138.12, 134.53, 129.40, 127.46, 120.80, 120.27, 116.34, 112.65, 55.54, 38.97, 19.22. HRMS (EI) *m/z* $[M+H]^+$ calc.: 281.1654; found: 281.1654

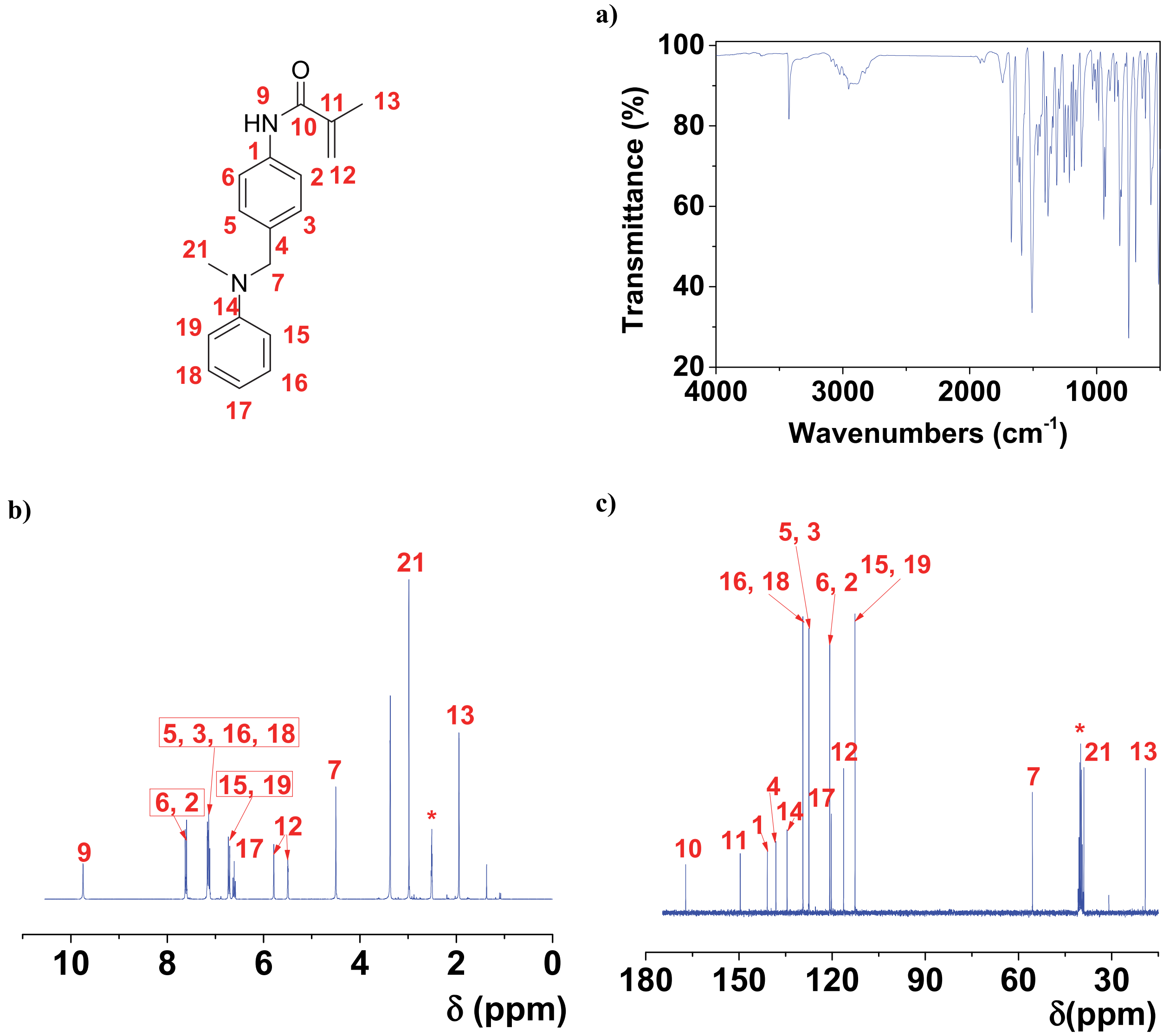

Figure S9 Characterization of MCM**2** by (a) FTIR, (b) ^{1}H NMR, and (c) ^{13}C NMR spectroscopies (* = solvent signal, DMSO-*d*6).

- ***Synthesis of tert-butyl 4-methacryloylpiperazine-1-carboxylate (9)***

A mixture of 1-Boc-piperazine (CAS 57260-71-6) (1 g, 5.37 mmol), methacrylic anhydride (802 µL, 828.2 mg, 5.37 mmol) and 50 mL of diethyl ether was stirred overnight in a pressured flask at 50 ºC. After that, the solvent was removed under reduced pressure. Yield: 95%. ^{1}H NMR (400 MHz, DMSO-d_6) δ (ppm) = 5.20 (s, 1H), 5.00 (s, 1H), 3.43 (s, 4H), 3.34 (s, 4H), 1.86 (s, 3H), 1.42 (s, 9H). ^{13}C NMR (75 MHz, DMSO-d_6) δ (ppm) = 170.47, 154.23, 140.45, 115.70, 79.61, 28.45, 18.52. HRMS (EI) *m/z* [M+H]$^+$ calc.: 255.1630; found: 255.1702.

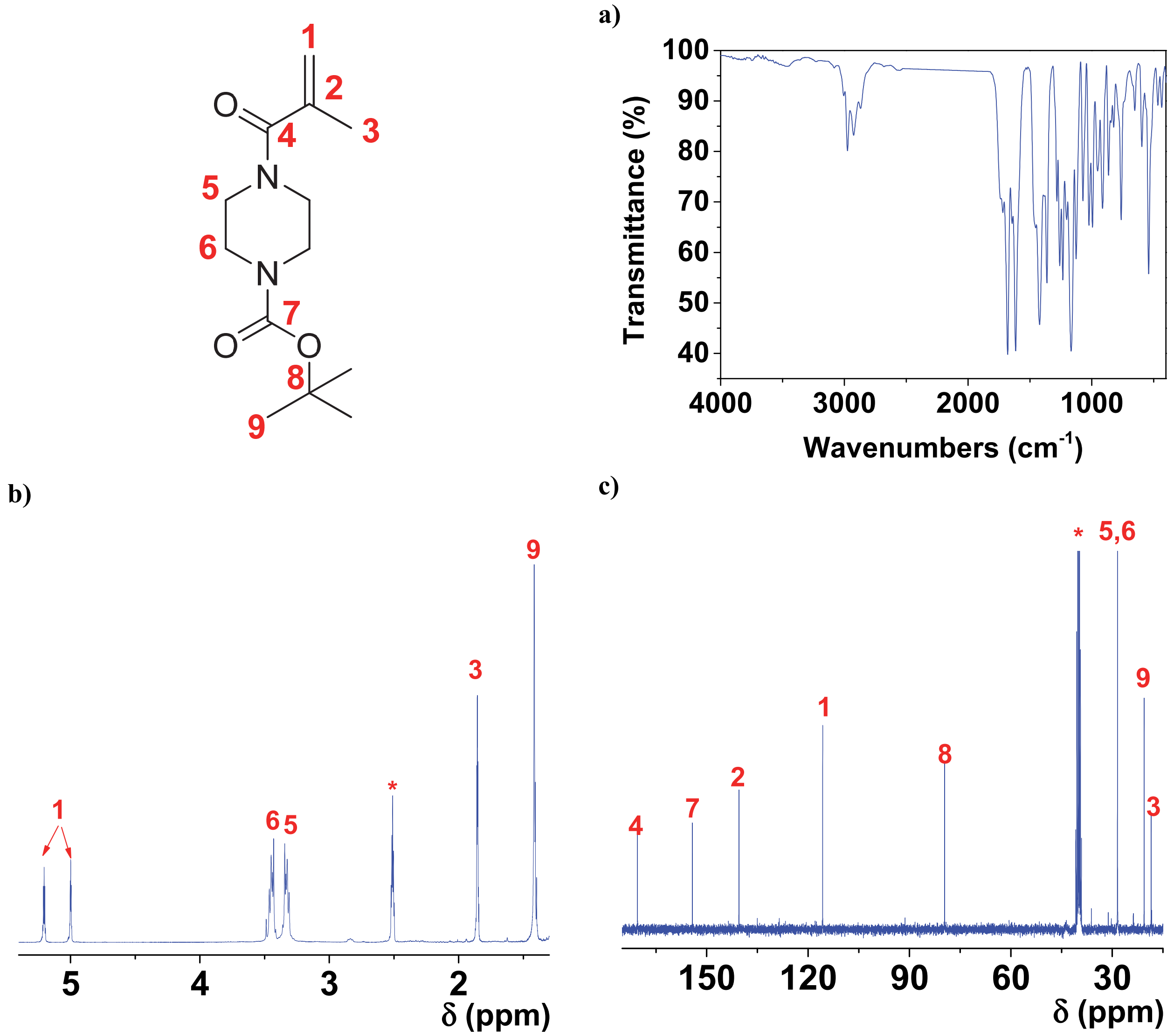

Figure S10. Characterization of MCM**2** by (a) FTIR, (b) ^{1}H NMR, and (c) ^{13}C NMR spectroscopies (* = solvent signal, DMSO-d_6).

- ***Synthesis of 2-methyl-1-(piperazin-1-yl)prop-2-en-1-one (10)***

A mixture tert-butyl 4-methacryloylpiperazine-1-carboxylate (9) (1.3 g, 5.1 mmol), 5mL of HCl (37%) and 10 mL of dioxane was stirred in a round-bottom flask at room temperature. After 30 minutes, the solvent was removed under reduced pressure. The solid was treated with 100 mL of saturated solution of K_2CO_3 (1 g/mL), and the solution was extracted with DCM (2 x 75 mL). Finally, the organic phase was dried over Na_2SO_4, filtered and the solvent was removed under reduced pressure. Yield: 95%. ^{1}H NMR (300 MHz, DMSO-d_6) δ (ppm) = 5.15 (s, 1H), 4.93 (s, 1H), 3.38 (s, 4H), 2.66 (s, 4H), 1.84 (s, 3H). ^{13}C NMR (75 MHz, DMSO-d_6) δ (ppm) = 170.18, 140.93, 114.92, 46.93, 20.68. HRMS (EI) *m/z* [M+H]$^+$ calc.: 155.1106; found: 155.1182.

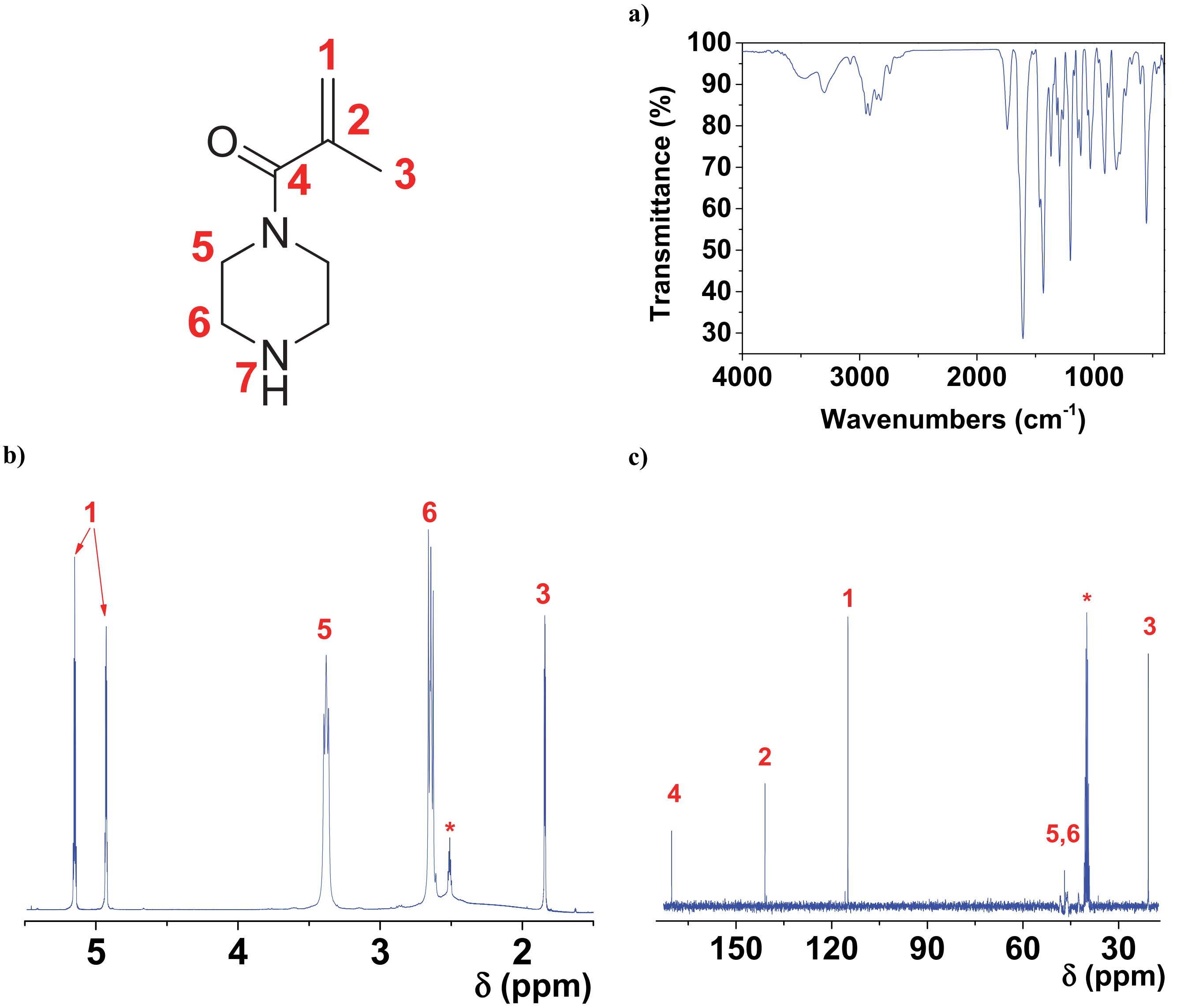

Figure S11. Characterization of MCM**2** by (a) FTIR, (b) ^{1}H NMR, and (c) ^{13}C NMR spectroscopies (* = solvent signal, DMSO-d_6).

- ***Synthesis of 5-(chloromethyl)quinolin-8-ol (11)***

Synthesis and characterization carried out as described by *Rbaa et al.*[4]

- ***Synthesis of 1-(4-((8-hydroxyquinolin-5-yl)methyl)piperazin-1-yl)-2-methylprop-2-en-1-one (MCAR4)***

A mixture of 5-(chloromethyl)quinolin-8-ol (11) (1.45 g, 7.46 mmol), 2-methyl-1-(piperazin-1-yl)prop-2-en-1-one (10) (1.15 g, 7.46 mmol), triethylamine (3.12 mL, 2.26 mg, 22.4 mmol) and 75 mL of DCM was stirred in round-bottom flask at room temperature for 15 minutes after the reagents were dissolved. After that, the solution was washed with brine (2 x 50 mL). Finally, the organic phase was dried over Na_2SO_4, filtered and the solvent removed under reduced pressure. Yield: 75.5 %. ^1H NMR (300 MHz, DMSO-d_6) δ (ppm) = 9.72 (s, 1H), 8.86 (s, 1H), 8.65 (s, 1H), 7.61 (s, 1H), 7.32 (s, 1H), 7.02 (s, 1H), 5.16 (s, 1H), 4.94 (s, 1H), 3.79 (s, 2H), 3.39 (s, 6H), 2.38 (s, 4H), 1.84 (s, 3H). ^{13}C NMR (75 MHz, DMSO-d_6) δ (ppm) =. 170.14, 147.99, 140.67, 139.56, 134.23, 129.61, 128.44, 123.89, 121.79, 115.28, 110.61, 59.91, 52.90, 46.67, 20.65. HRMS (EI) *m/z* $[M+H]^+$ calc.: 312.1634; found: 312.1710.

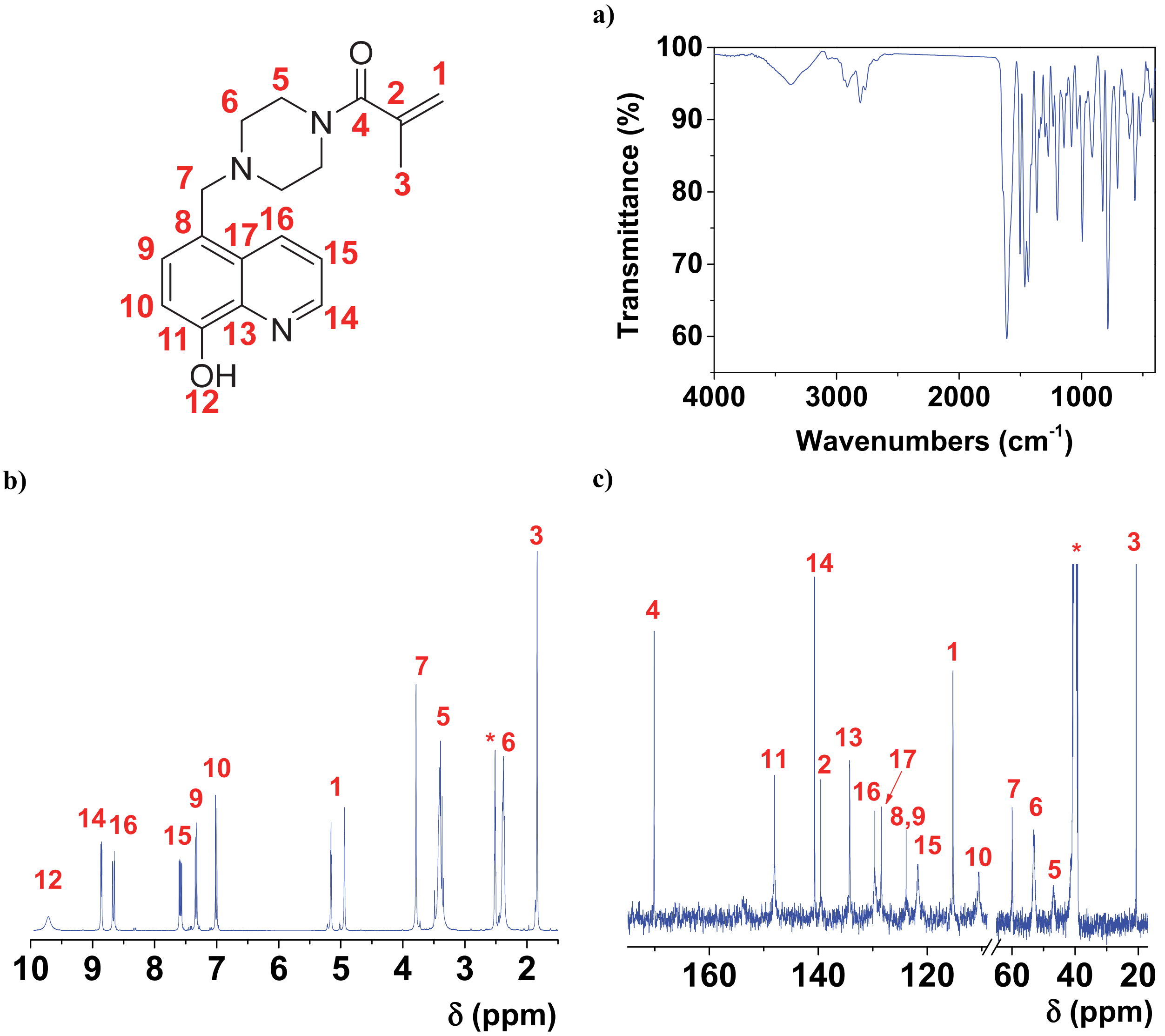

Figure S12. Characterization of MCM**2** by (a) FTIR, (b) ^1H NMR, and (c) ^{13}C NMR spectroscopies (* = solvent signal, DMSO-d_6).

S3. Characterization of sensory polymers

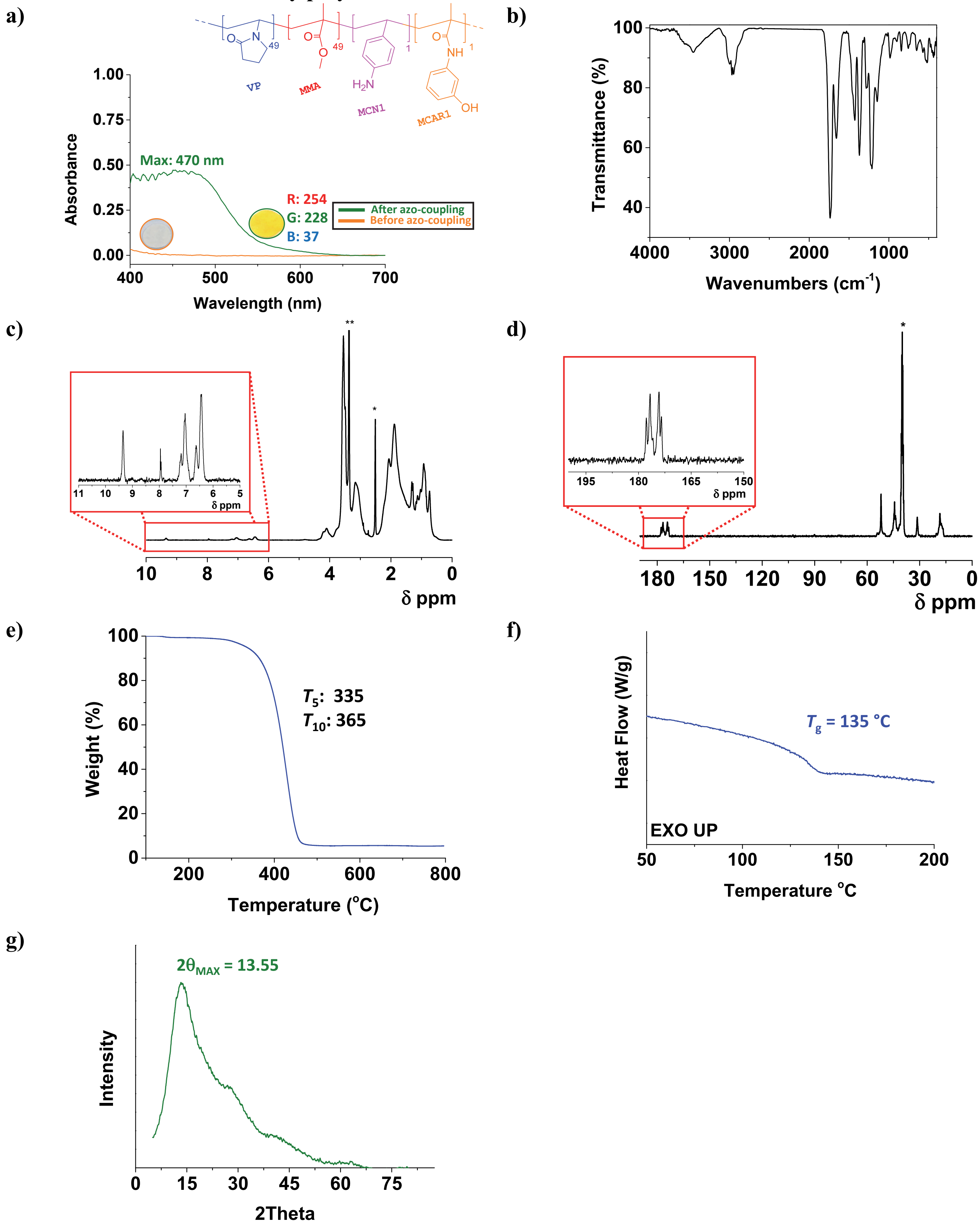

Figure S13. Polymer characterization by (**a**) UV-Vis spectrometry, spectra of the coated paper before and after azo-coupling reaction, including polymer´s formula and real photographs of coated discs. (**b**) FT-IR, Figure shows the IR spectra of the polymer (**c**) 1H RMN spectra (*=DMSO-d_6, **=H_2O). (**d**) 13C RMN (*=DMSO-d_6). (**e**) Thermogravimetric curves at 10°C·min^{-1} under nitrogen atmosphere showing T_5 and T_{10} temperatures. (**f**) DSC curve at a heating rate of 20°C·min^{-1} under nitrogen atmosphere showing Tg value. (**g**) PXRD spectra showing $2\theta_{MAX}$.

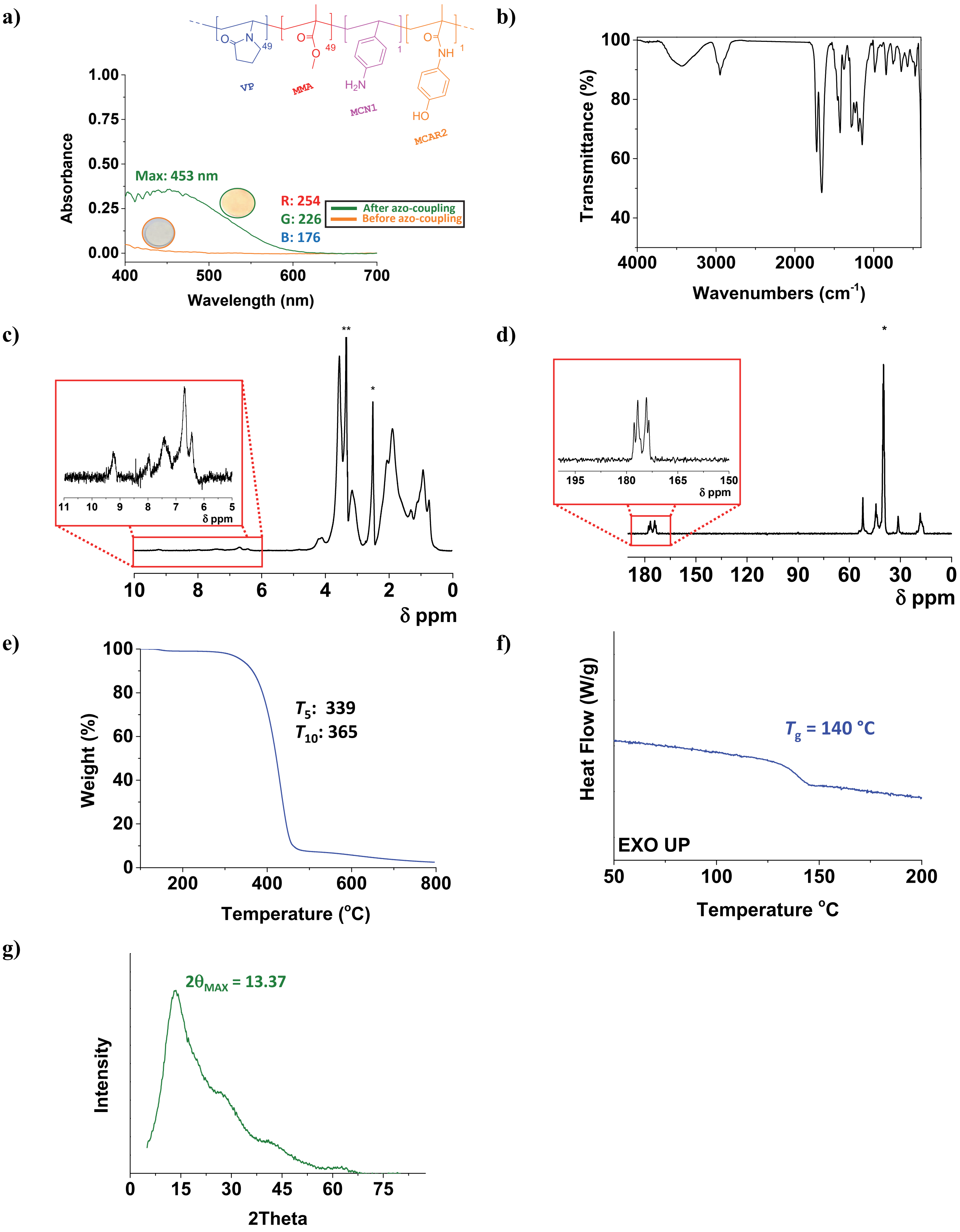

Figure S14. Polymer characterization by (**a**) UV-Vis spectrometry, spectra of the coated paper before and after azo-coupling reaction, including polymer´s formula and real photographs of coated discs. (**b**) FT-IR, Figure shows the IR spectra of the polymer (**c**) ^{1}H RMN spectra (*=DMSO-d_6, **=H_2O). (**d**) ^{13}C RMN (*=DMSO-d_6). (**e**) Thermogravimetric curves at 10°C·min^{-1} under nitrogen atmosphere showing T_5 and T_{10} temperatures. (**f**) DSC curve at a heating rate of 20°C·min^{-1} under nitrogen atmosphere showing Tg value. (**g**) PXRD spectra showing $2\theta_{MAX}$.

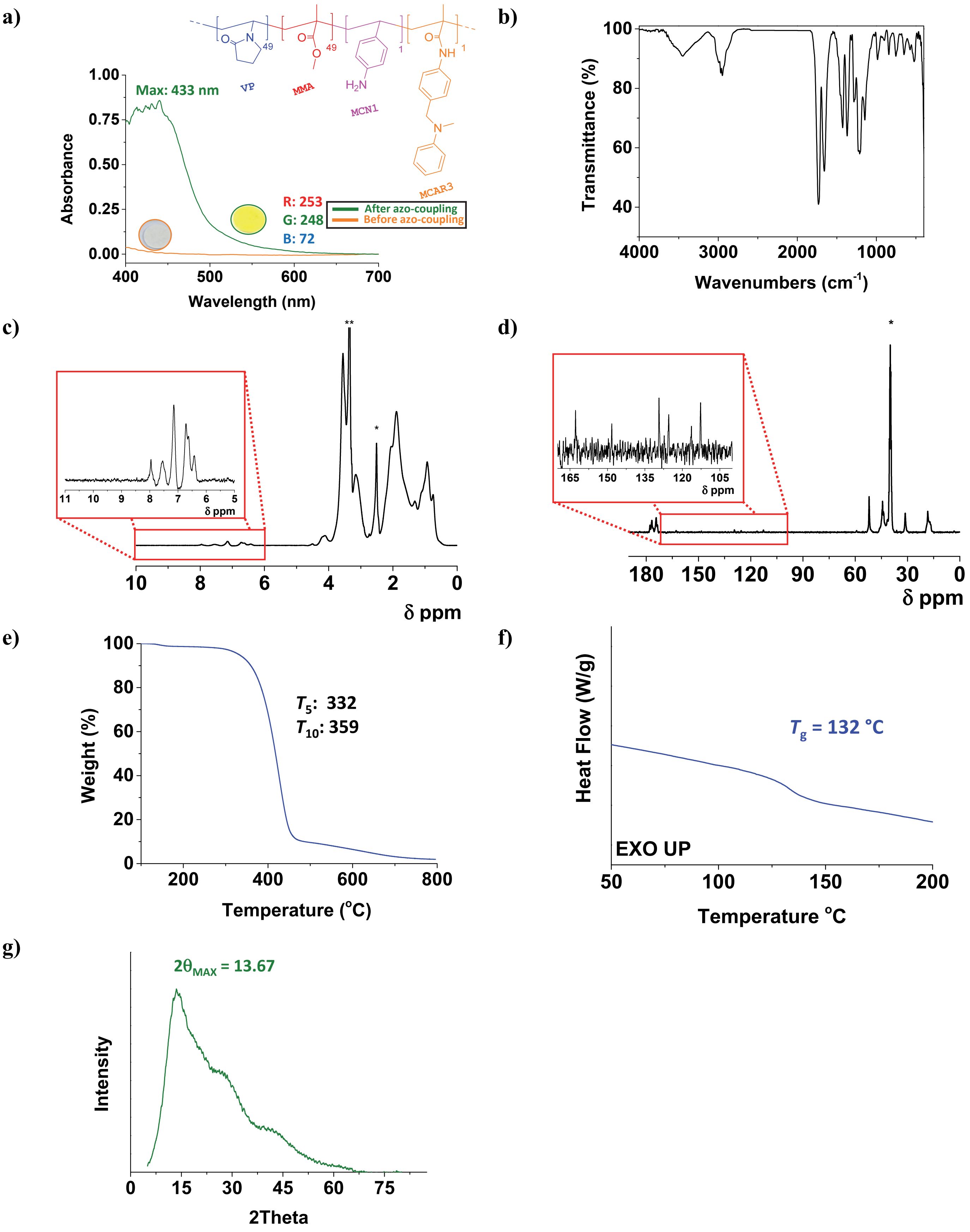

Figure S15. Polymer characterization by (**a**) UV-Vis spectrometry, spectra of the coated paper before and after azo-coupling reaction, including polymer´s formula and real photographs of coated discs. (**b**) FT-IR, Figure shows the IR spectra of the polymer (**c**) ^{1}H RMN spectra (*=DMSO-d_6, **=H_2O). (**d**) ^{13}C RMN (*=DMSO-d_6). (**e**) Thermogravimetric curves at 10°C·min^{-1} under nitrogen atmosphere showing T_5 and T_{10} temperatures. (**f**) DSC curve at a heating rate of 20°C·min^{-1} under nitrogen atmosphere showing Tg value. (**g**) PXRD spectra showing $2\theta_{MAX}$.

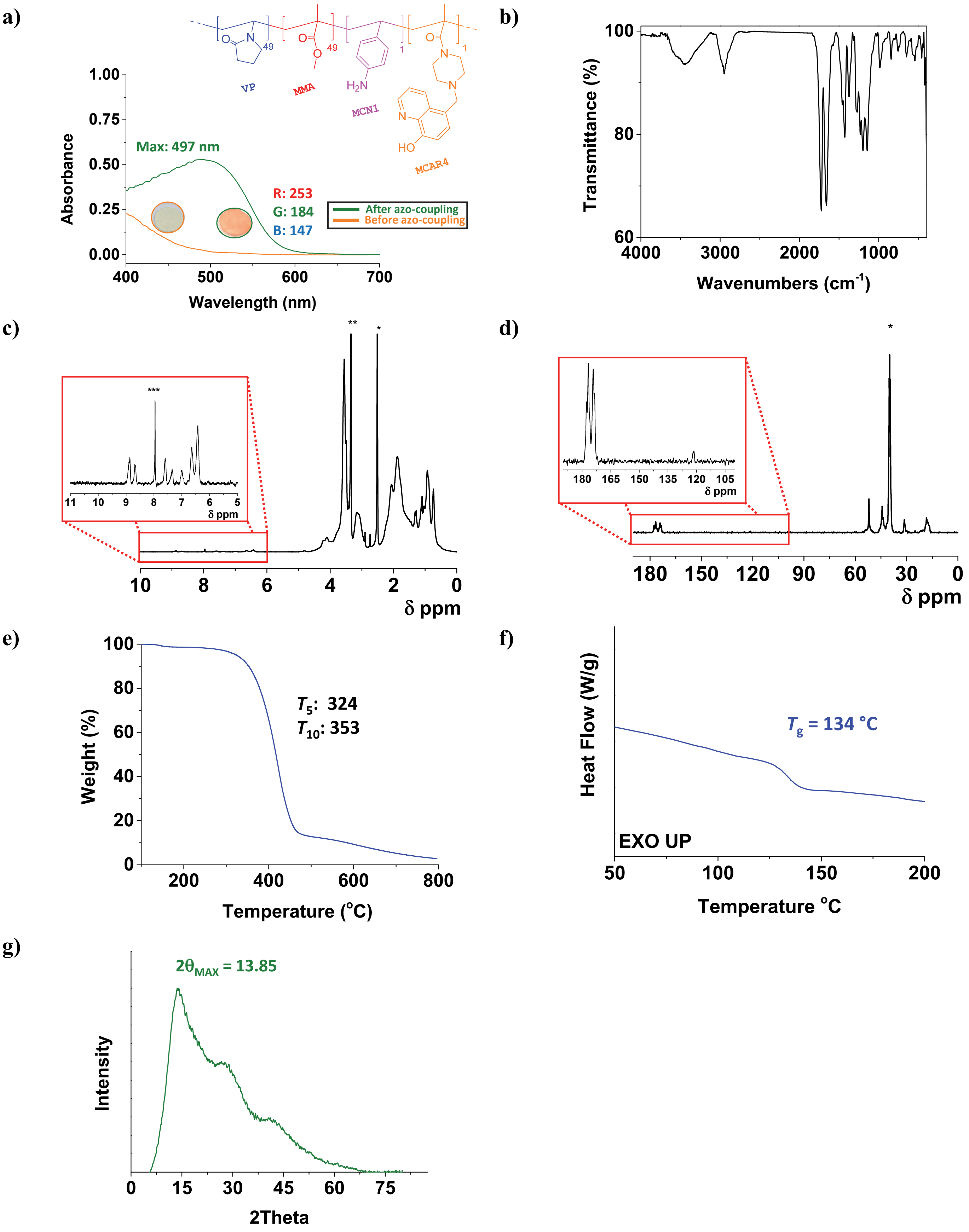

Figure S16. Polymer characterization by (**a**) UV-Vis spectrometry, spectra of the coated paper before and after azo-coupling reaction, including polymer´s formula and real photographs of coated discs. (**b**) FT-IR, Figure shows the IR spectra of the polymer (**c**) ^{1}H RMN spectra (*=DMSO-δ_6, **=H_2O). (**d**) ^{13}C RMN (*=DMSO-δ_6). (**e**) Thermogravimetric curves at 10°C·min^{-1} under nitrogen atmosphere showing T_5 and T_{10} temperatures. (**f**) DSC curve at a heating rate of 20°C·min^{-1} under nitrogen atmosphere showing Tg value. (**g**) PXRD spectra showing $2\theta_{MAX}$.

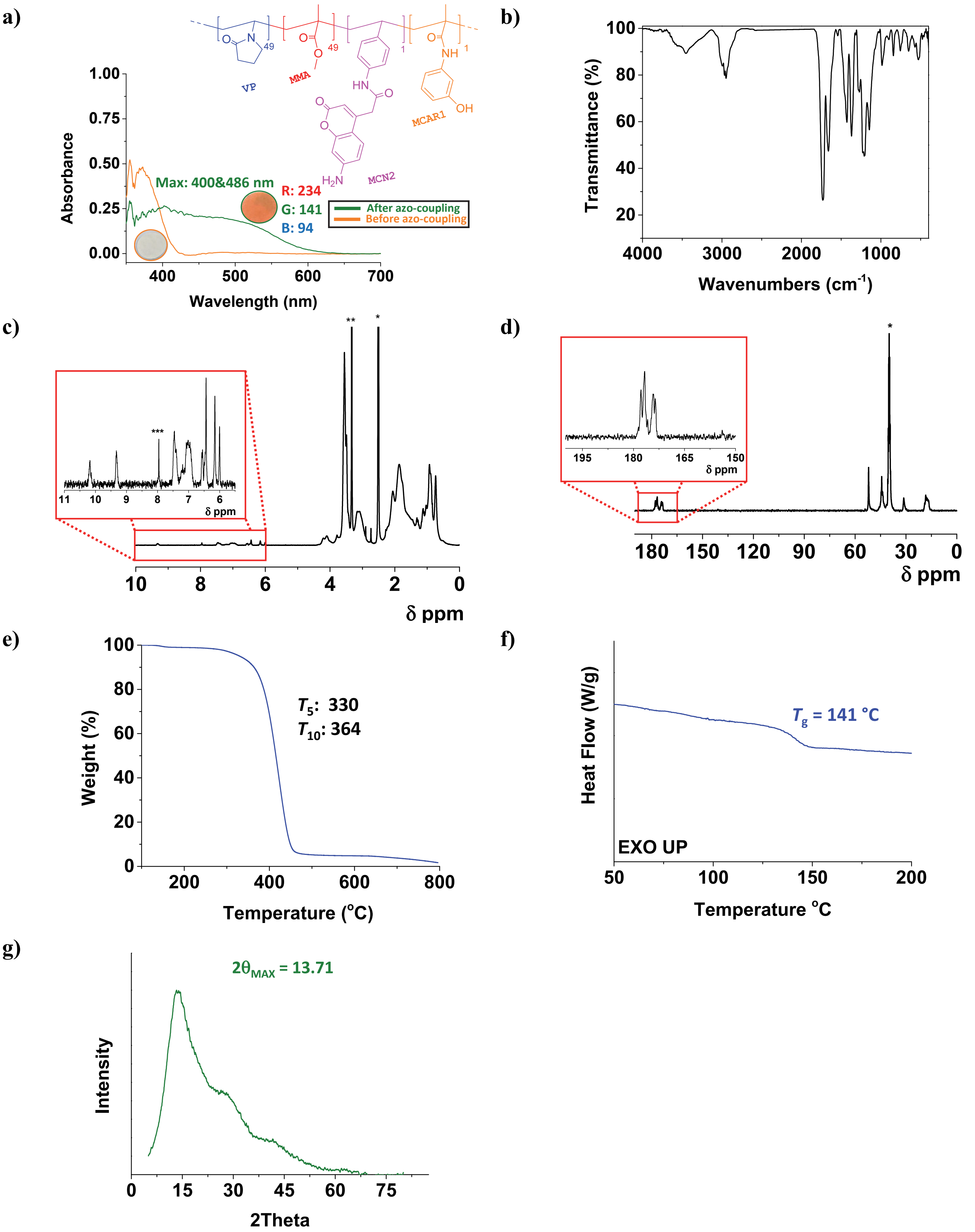

Figure S17. Polymer characterization by (**a**) UV-Vis spectrometry, spectra of the coated paper before and after azo-coupling reaction, including polymer´s formula and real photographs of coated discs. (**b**) FT-IR, Figure shows the IR spectra of the polymer (**c**) ^{1}H RMN spectra (*=DMSO-d$_6$, **=H_2O). (**d**) ^{13}C RMN (*=DMSO-d$_6$). (**e**) Thermogravimetric curves at 10°C·min^{-1} under nitrogen atmosphere showing T_5 and T_{10} temperatures. (**f**) DSC curve at a heating rate of 20 °C·min^{-1} under nitrogen atmosphere showing Tg value. (**g**) PXRD spectra showing $2\theta_{MAX}$.

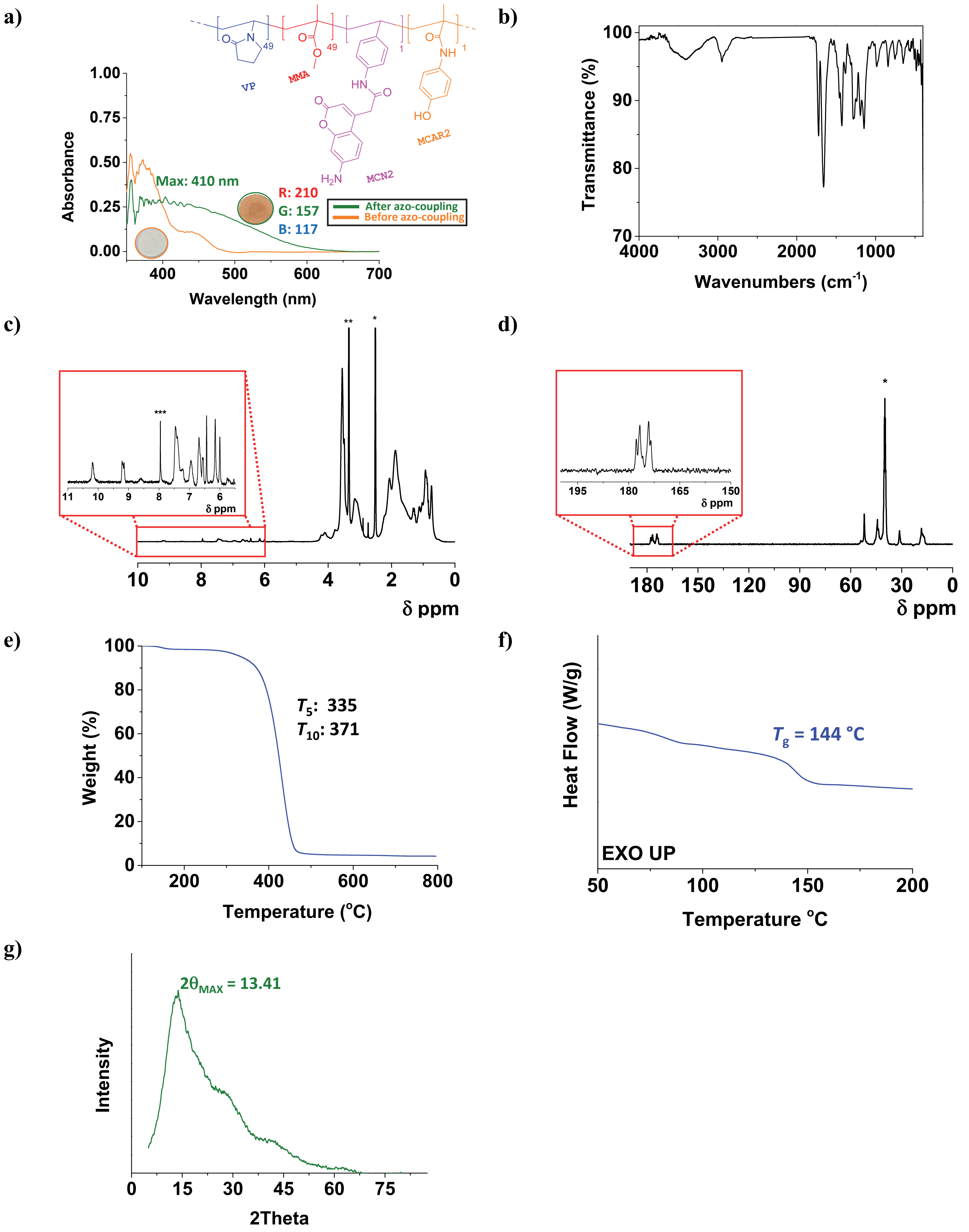

Figure S18. Polymer characterization by (**a**) UV-Vis spectrometry, spectra of the coated paper before and after azo-coupling reaction, including polymer´s formula and real photographs of coated discs. (**b**) FT-IR, Figure shows the IR spectra of the polymer (**c**) ^{1}H RMN spectra (*=DMSO-d$_6$, **=H_2O). (**d**) ^{13}C RMN (*=DMSO-d$_6$). (**e**) Thermogravimetric curves at 10°C·min^{-1} under nitrogen atmosphere showing T_5 and T_{10} temperatures. (**f**) DSC curve at a heating rate of 20°C·min^{-1} under nitrogen atmosphere showing Tg value. (**g**) PXRD spectra showing 2θ$_{MAX}$.

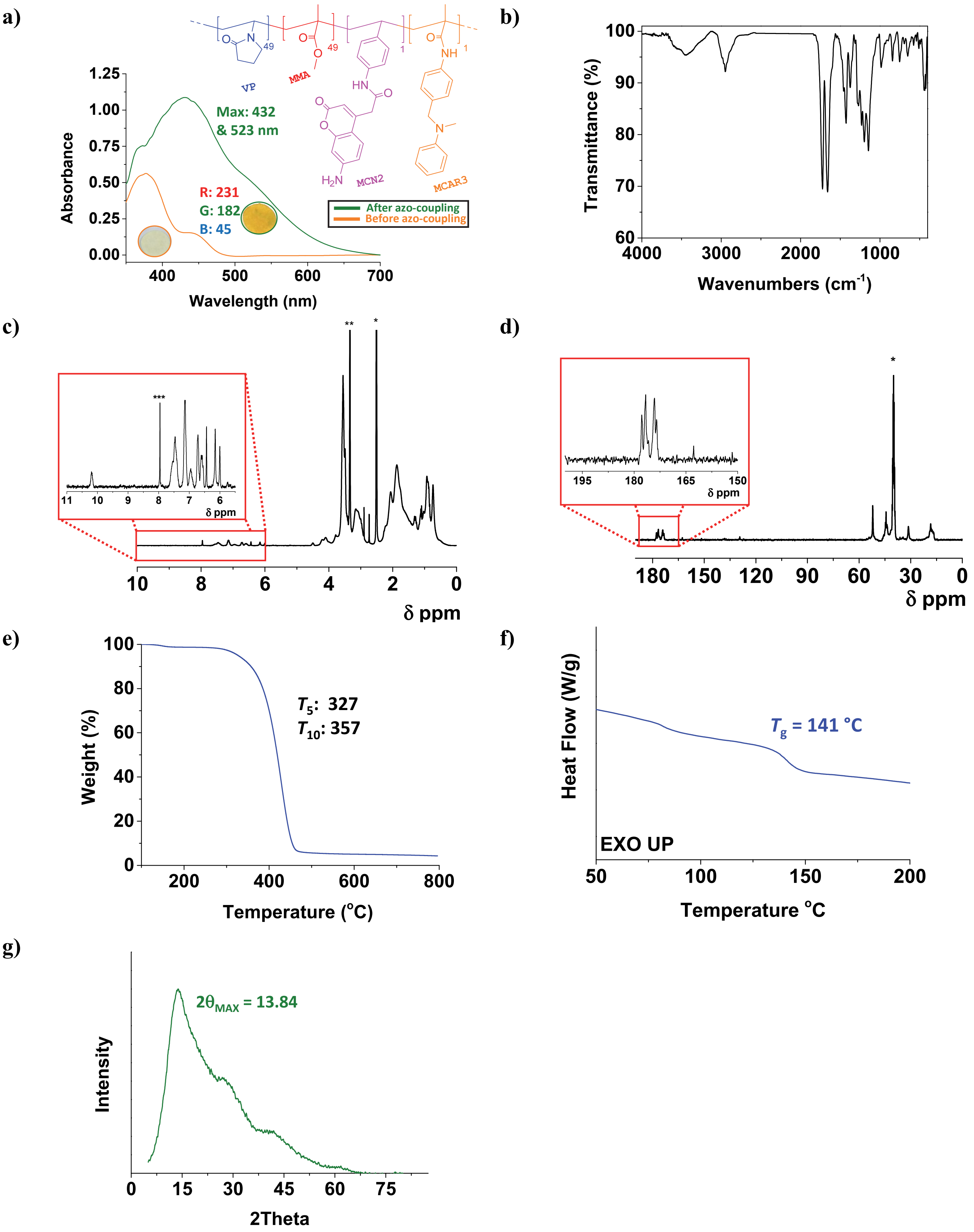

Figure S19. Polymer characterization by (**a**) UV-Vis spectrometry, spectra of the coated paper before and after azo-coupling reaction, including polymer´s formula and real photographs of coated discs. (**b**) FT-IR, Figure shows the IR spectra of the polymer (**c**) ^{1}H RMN spectra (*=DMSO-d$_6$, **=H_2O). (**d**) ^{13}C RMN (*=DMSO-d$_6$). (**e**) Thermogravimetric curves at 10°C·min^{-1} under nitrogen atmosphere showing T_5 and T_{10} temperatures. (**f**) DSC curve at a heating rate of 20°C·min^{-1} under nitrogen atmosphere showing Tg value. (**g**) PXRD spectra showing 2θ$_{MAX}$.

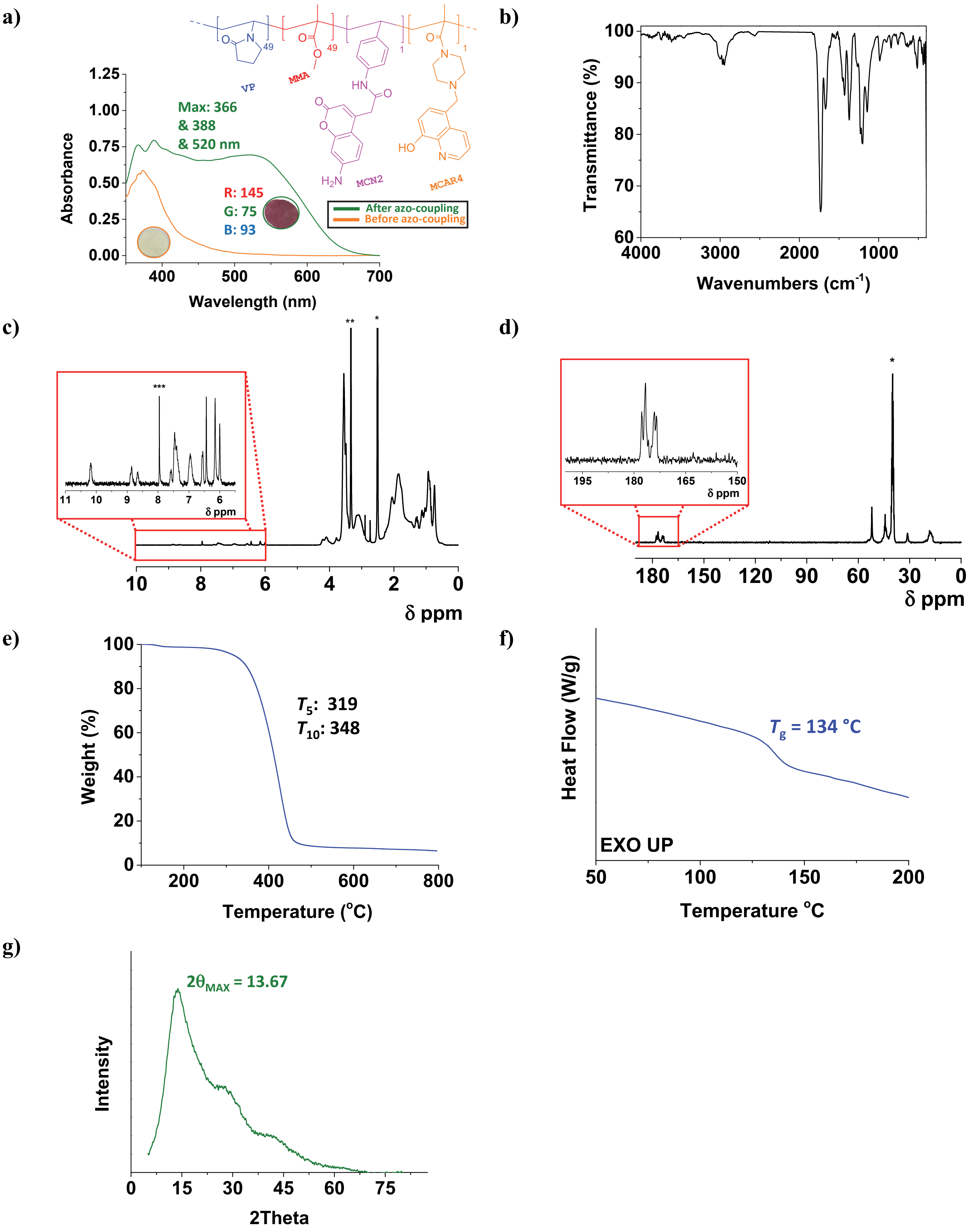

Figure S20. Polymer characterization by (**a**) UV-Vis spectrometry, spectra of the coated paper before and after azo-coupling reaction, including polymer´s formula and real photographs of coated discs. (**b**) FT-IR, Figure shows the IR spectra of the polymer (**c**) ^{1}H RMN spectra (*=DMSO-d$_6$, **=H_2O). (**d**) ^{13}C RMN (*=DMSO-d$_6$). (**e**) Thermogravimetric curves at 10°C·min^{-1} under nitrogen atmosphere showing T_5 and T_{10} temperatures. (**f**) DSC curve at a heating rate of 20°C·min^{-1} under nitrogen atmosphere showing Tg value. (**g**) PXRD spectra showing 2θ$_{MAX}$.

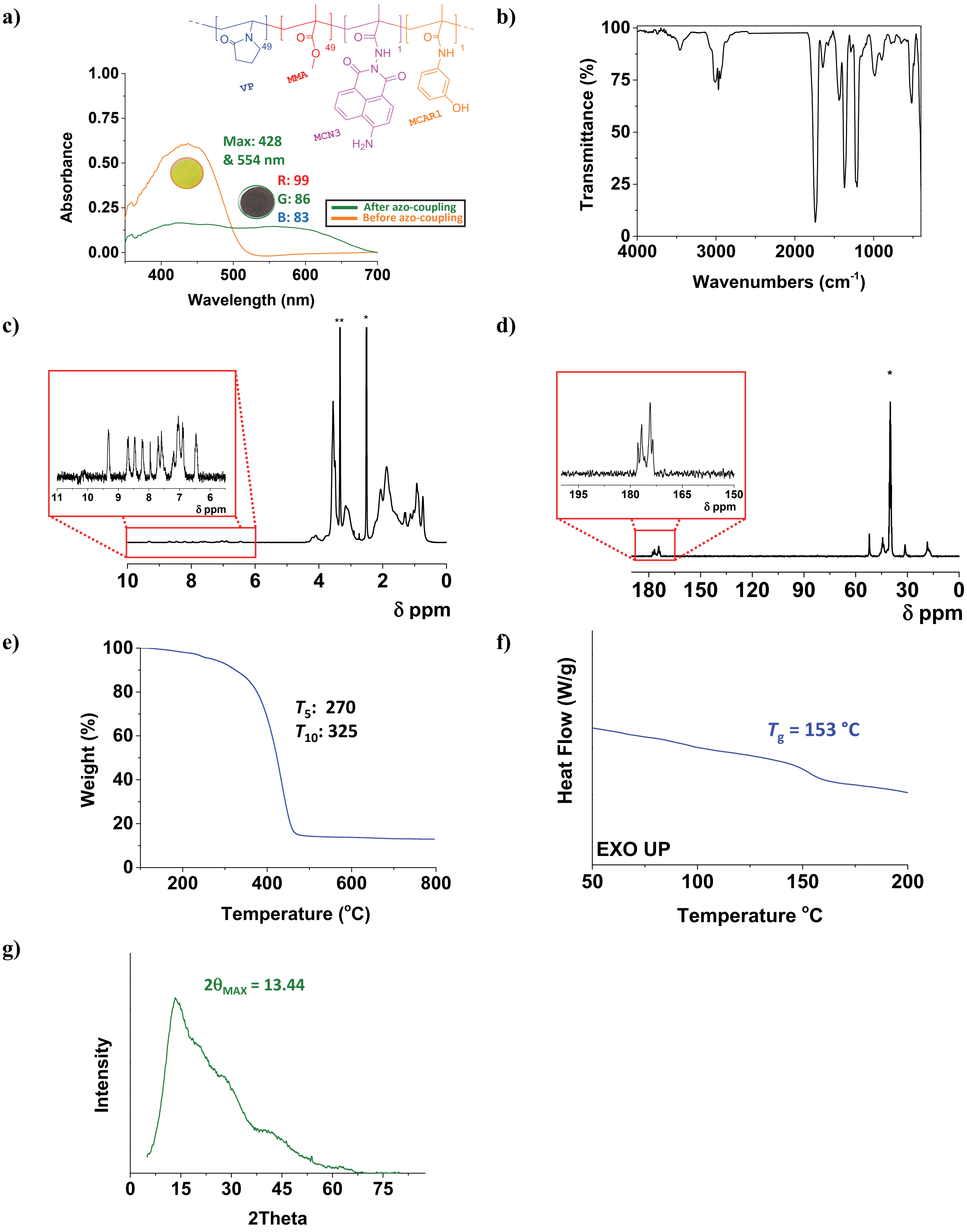

Figure S21. Polymer characterization by (**a**) UV-Vis spectrometry, spectra of the coated paper before and after azo-coupling reaction, including polymer´s formula and real photographs of coated discs. (**b**) FT-IR, Figure shows the IR spectra of the polymer (**c**) ^{1}H RMN spectra (*=DMSO-d$_6$, **=H_2O). (**d**) ^{13}C RMN (*=DMSO-d$_6$). (**e**) Thermogravimetric curves at 10°C·min^{-1} under nitrogen atmosphere showing T_5 and T_{10} temperatures. (**f**) DSC curve at a heating rate of 20°C·min^{-1} under nitrogen atmosphere showing Tg value. (**g**) PXRD spectra showing 2θ$_{MAX}$.

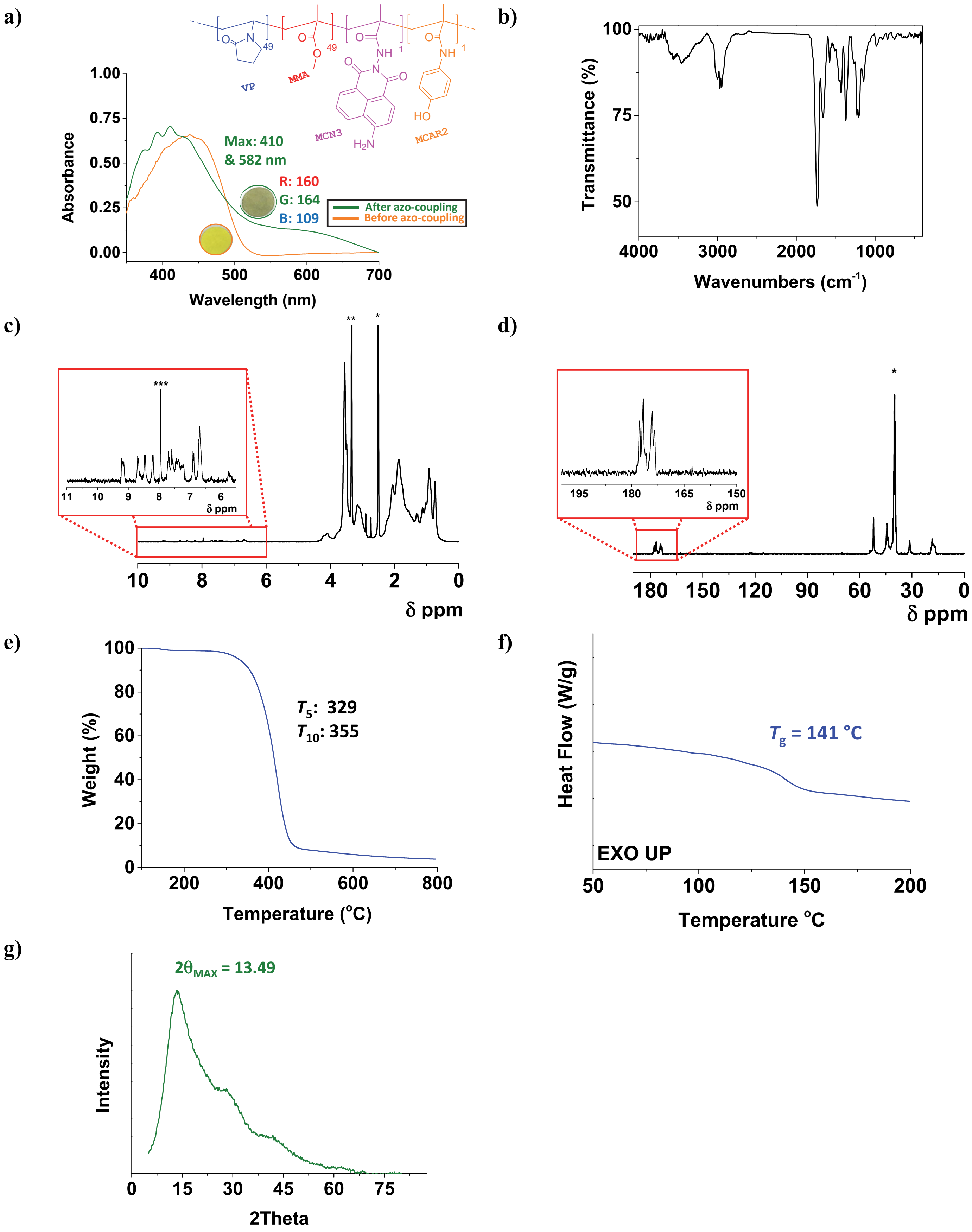

Figure S22. Polymer characterization by (**a**) UV-Vis spectrometry, spectra of the coated paper before and after azo-coupling reaction, including polymer´s formula and real photographs of coated discs. (**b**) FT-IR, Figure shows the IR spectra of the polymer (**c**) ^{1}H RMN spectra (*=DMSO-d$_6$, **=H_2O). (**d**) ^{13}C RMN (*=DMSO-d$_6$). (**e**) Thermogravimetric curves at 10°C·min^{-1} under nitrogen atmosphere showing T_5 and T_{10} temperatures. (**f**) DSC curve at a heating rate of 20°C·min^{-1} under nitrogen atmosphere showing Tg value. (**g**) PXRD spectra showing 2θ$_{MAX}$.

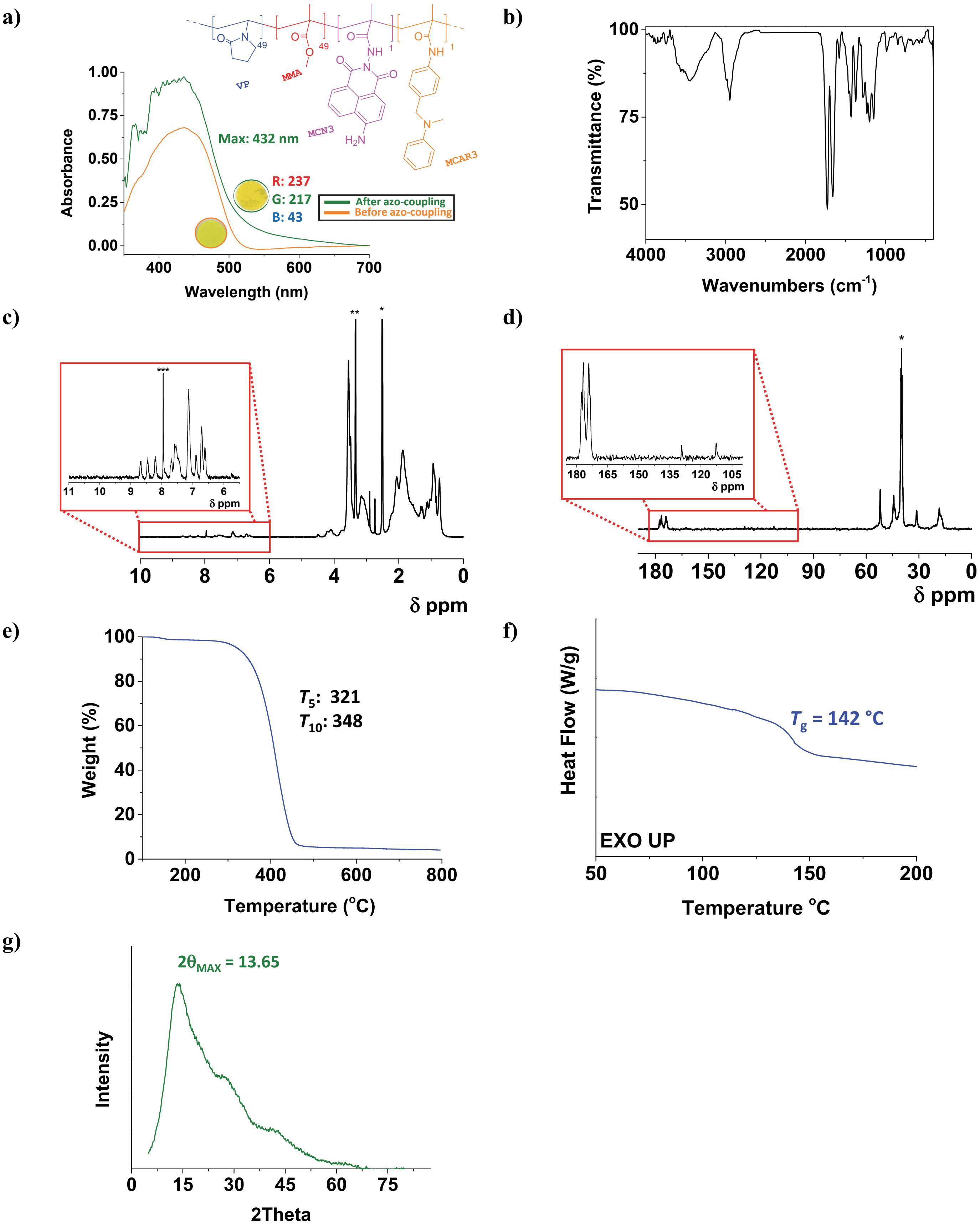

Figure S23. Polymer characterization by (**a**) UV-Vis spectrometry, spectra of the coated paper before and after azo-coupling reaction, including polymer´s formula and real photographs of coated discs. (**b**) FT-IR, Figure shows the IR spectra of the polymer (**c**) ^{1}H RMN spectra (*=DMSO-d_6, **=H_2O). (**d**) ^{13}C RMN (*=DMSO-d_6). (**e**) Thermogravimetric curves at 10°C·min^{-1} under nitrogen atmosphere showing T_5 and T_{10} temperatures. (**f**) DSC curve at a heating rate of 20°C·min^{-1} under nitrogen atmosphere showing Tg value. (**g**) PXRD spectra showing $2\theta_{MAX}$.

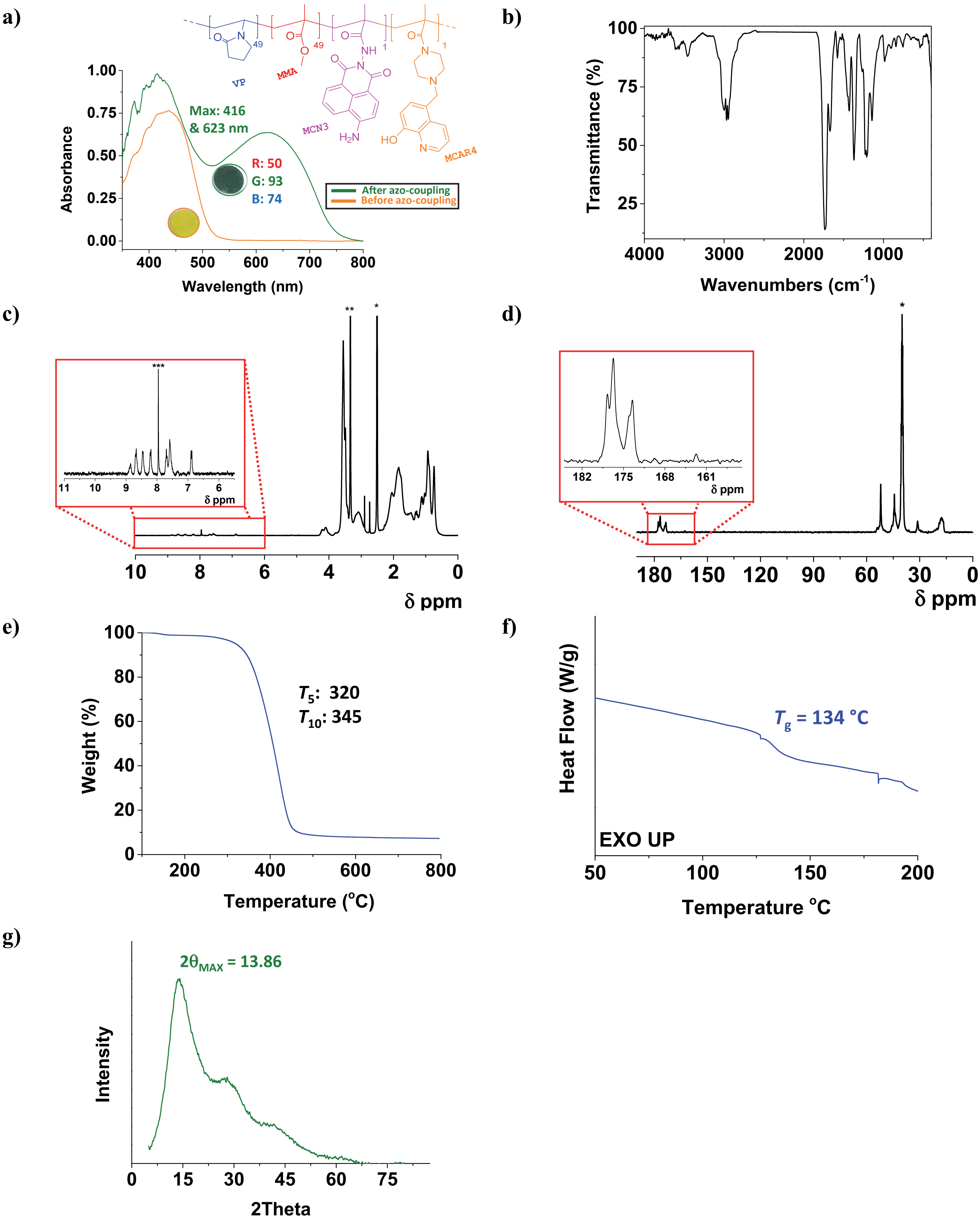

Figure S24. Polymer characterization by (**a**) UV-Vis spectrometry, spectra of the coated paper before and after azo-coupling reaction, including polymer´s formula and real photographs of coated discs. (**b**) FT-IR, Figure shows the IR spectra of the polymer (**c**) ^{1}H RMN spectra (*=DMSO-d$_6$, **=H_2O). (**d**) ^{13}C RMN (*=DMSO-d$_6$). (**e**) Thermogravimetric curves at 10°C·min^{-1} under nitrogen atmosphere showing T_5 and T_{10} temperatures. (**f**) DSC curve at a heating rate of 20°C·min^{-1} under nitrogen atmosphere showing Tg value. (**g**) PXRD spectra showing 2θ$_{MAX}$.

References

(1) Kellner, S.; Seidu-Larry, S.; Burhenne, J.; Motorin, Y.; Helm, M. A Multifunctional Bioconjugate Module for Versatile Photoaffinity Labeling and Click Chemistry of RNA. *Nucleic Acids Res.* **2011**, *39* (16), 7348–7360. https://doi.org/10.1093/nar/gkr449.

(2) Thorson, M. K.; Van Wagoner, R. M.; Harper, M. K.; Ireland, C. M.; Majtan, T.; Kraus, J. P.; Barrios, A. M. Marine Natural Products as Inhibitors of Cystathionine Beta-Synthase Activity. *Bioorganic Med. Chem. Lett.* **2015**, *25* (5), 1064–1066. https://doi.org/10.1016/j.bmcl.2015.01.013.

(3) GUEMBE-GARCÍA, M.; GONZÁLEZ-CEBALLOS, L.; FERNÁNDEZ-MUIÑO, M. A.; SANCHO, M. T.; OSÉS, S. M.; IBEAS, S.; ROVIRA, J.; MELERO, B.; REPRESA, C.; GARCÍA, J. M.; VALLEJOS, S. Rapid and Inexpensive Detection of Nitrites in Processed Meat Using a Colorimetric Polymer Sensor and a Mobile App. *Results under Publ. Process.* **2021**.

(4) Rbaa, M.; Fardioui, M.; Verma, C.; Abousalem, A. S.; Galai, M.; Ebenso, E. E.; Guedira, T.; Lakhrissi, B.; Warad, I.; Zarrouk, A. 8-Hydroxyquinoline Based Chitosan Derived Carbohydrate Polymer as Biodegradable and Sustainable Acid Corrosion Inhibitor for Mild Steel: Experimental and Computational Analyses. *Int. J. Biol. Macromol.* **2020**, *155*, 645–655. https://doi.org/10.1016/j.ijbiomac.2020.03.200.
